# Calcium-Dependent Protein Kinase GhCDPK28 Was Dentified and Involved in Verticillium Wilt Resistance in Cotton

**DOI:** 10.3389/fpls.2021.772649

**Published:** 2021-12-15

**Authors:** Yajie Wu, Lei Zhang, Jinglong Zhou, Xiaojian Zhang, Zili Feng, Feng Wei, Lihong Zhao, Yalin Zhang, Hongjie Feng, Heqin Zhu

**Affiliations:** ^1^Zhengzhou Research Base, State Key Laboratory of Cotton Biology, Zhengzhou University, Zhengzhou, China; ^2^School of Agricultural Sciences, Zhengzhou University, Zhengzhou, China; ^3^State Key Laboratory of Cotton Biology, Institute of Cotton Research of CAAS, Anyang, China

**Keywords:** cotton, Verticillium wilt, *V. dahliae*, resistance gene, calcium-dependent protein kinase

## Abstract

*Verticillium dahliae* is a soil-borne fungus that causes vascular wilt through the roots of plants. Verticillium wilt caused by *V. dahliae* is one of the main diseases in cotton producing areas of the world, resulting in huge economic losses. Breeding resistant varieties is the most economical and effective method to control Verticillium wilt. Calcium-dependent protein kinases (CDPKs) play a pivotal role in plant innate immunity, including regulation of oxidative burst, gene expression as well as hormone signal transduction. However, the function of cotton CDPKs in response to *V. dahliae* stress remains unexplored. In this study, 96, 44 and 57 CDPKs were identified from *Gossypium hirsutum*, *Gossypium raimondii* and *Gossypium arboretum*, respectively. Phylogenetic analysis showed that these CDPKs could be divided into four branches. All GhCDPKs of the same clade are generally similar in gene structure and conserved domain arrangement. Cis-acting elements related to hormones, stress response, cell cycle and development were predicted in the promoter region. The expression of GhCDPKs could be regulated by various stresses. Gh_D11G188500.1 and Gh_A11G186100.1 was up-regulated under Vd0738 and Vd991 stress. Further phosphoproteomics analysis showed that Gh_A11G186100.1 (named as GhCDPK28-6) was phosphorylated under the stress of *V. dahliae*. Knockdown of *GhCDPK28-6* expression, the content of reactive oxygen species was increased, a series of defense responses were enhanced, and the sensitivity of cotton to *V. dahliae* was reduced. Moreover, overexpression of *GhCDPK28-6* in *Arabidopsis thaliana* weakened the resistance of plants to this pathogen. Subcellular localization revealed that GhCDPK28-6 was localized in the cell membrane. We also found that GhPBL9 and GhRPL12C may interact with GhCDPK28-6. These results indicate that *GhCDPK28-6* is a potential molecular target for improving resistance to Verticillium wilt in cotton. This lays a foundation for breeding disease-resistant varieties.

## Introduction

Cotton is primary source of natural fiber used in the textile industry, which is an important crop in the world (Liu et al., 2014). However, numerous biotic and abiotic stress are the main factors contributing to the yield reduction. Among them, Verticillium wilt caused by *Verticillium dahliae* is one of the main diseases in cotton producing areas of the world, resulting in huge economic losses (Shaban et al., 2018). *V. dahliae* is a soil-borne fungus that reproduces asexually and causes vascular wilt through the roots of plants (Atallah et al., 2011). The fungus can form dormant structures called microsclerotia that can survive in the soil for years without a host and infect subsequent crops (Klosterman et al., 2009; Gong et al., 2018). Currently, no fungicide can effectively control Verticillium wilt once the plant has been infected (Fradin and Thomma, 2006). The selection and breeding of disease-resistant varieties is the fundamental measure of disease control worldwide (Yang et al., 2020). The key factors in the resistance and regulation mechanism of Verticillium wilt resistance in cotton are still poorly understood (Shaban et al., 2018). Therefore, it is very necessary to identify Verticillium wilt resistance genes in cotton germplasm and integrate them into high-quality cotton varieties (Li et al., 2019b).

Plants have evolved a multi-layered surveillance system, and innate immunity is the first line of inducible defense against pathogens (Nurnberger et al., 2004). There are two layers of innate immune system that plants use to defense against microbial infection: pattern-triggered immunity (PTI) and effector-triggered immunity (ETI; Li et al., 2016). Pattern recognition receptors (PRRs) that recognize pathogen- or microbial-associated molecular patterns (PAMPs/MAMPs) and endogenous damage-associated molecular patterns (DAMPs) trigger PTI (Macho and Zipfel, 2014). Molecular signatures of pathogens are detected by PRRs, triggering a burst of reactive oxygen species (ROS), secondary metabolite production and calcium ion (Ca^2+^) influx into the cytosol (Alves et al., 2021). ROS and nitric oxide (NO) are key signaling molecules involved in various developmental and stress responses in plants (Kim et al., 2017). Callose deposits and lignin accumulation form a barrier in the early stages of pathogen invasion and are markers of a plant’s defense response (Luna et al., 2011; Tang et al., 2019). Hormone mediated signaling is one of the important defense mechanisms of cotton against *V. dahliae*. Among them, salicylic acid (SA), jasmonic acid (JA) and ethylene (ET) play important roles in post-infection stress signaling pathways such as ROS and MAP kinases (Shaban et al., 2018).

In recent years, important advances have been made in genomics, transcriptomics and proteomics. Many genes associated with *V. dahliae* resistance in cotton have been reported. Many transcription factors (TFs) are involved in plant defense against pathogen attack, including WRKY, NAC, bHLH, bZIP, ERF/AP2, and MYB family members (Singh et al., 2002; Buscaill and Rivas, 2014). *V. dahliae* infection causes *GhWAKL* expression in cotton and responds to SA application. *GhWAKL* overexpression in *Arabidopsis thaliana* enhances its resistance to pathogens (Feng et al., 2021). The expression of *GhMYB108* in cotton plants is induced by *V. dahliae* infection and responds to the application of defense signaling molecules such as SA, JA and ET (Cheng et al., 2016). *GbNAC1* is involved in the positive regulation of Verticillium wilt resistance (Wang et al., 2016b). Overexpression of *GhbHLH171* in cotton activated JA synthesis and signaling pathways, and improved cotton tolerance to *V. dahliae* (He et al., 2018). *GbERF2* plays an important role in the response of cotton to ethylene stress and pathogen invasion (Zuo et al., 2007). Receptor-like protein kinase (RLKs), Cytochrome P450 (CYPs) and (+)-delta-cadinene synthase are key regulatory gene families involved in defense response (Tan et al., 2000; Zhang et al., 2017). The function of genes was identified by Virus-induced gene silencing (VIGS) technology, and it was found that *GHMKK2*, *GhVe1*, *GbCAD1* and *GhNDR1* genes play an important role in cotton Verticillium wilt resistance (Gao et al., 2011, 2013a,b).

Ca^2+^ is a ubiquitous second messenger in plant cells and participates in many signaling pathways (Lecourieux et al., 2006). As an essential conserved medium in plant defense response, Ca^2+^can respond to abiotic stresses and microbial inducers (Hu et al., 2020). Calmodulins/calmodulin-like proteins, calcineurin B-like proteins and calcium-dependent protein kinases (CDPKs) are three main types of Ca^2+^ sensors in plants (Harper et al., 1991). CDPKs are serine/threonine protein kinases, which only exist in plants and some protozoa (Boudsocq et al., 2012). CDPK proteins contain four characteristic domains: the N-terminal variable region, the Ser/Thr kinase catalytic domain, the autoregulatory/autoinhibitory domain and the calmodulin-like domain (Ye et al., 2009). CDPKs play a pivotal role in plant innate immunity, including regulation of oxidative burst, gene expression as well as hormone signal transduction (Liese and Romeis, 2013). During pathogen infection, CDPKs can be activated by calcium to induce conformational changes and the kinase activity, leading to CDPK autophosphorylation and target substrate phosphorylation (Cheng et al., 2002; Li et al., 2016). CDPK has a highly variable N-terminal domain, containing myristoylation or palmitoylation sites, both of which are related to the membrane localization of CDPK (Ye et al., 2009; Matschi et al., 2015). Different Ca^2+^ signals, CDPK activation kinetics and CDPK targets may be the reasons for the changes in the specificity, amplitude and intensity of PTI and ETI immune gene transcription (Li et al., 2016). 34 CDPKs have been identified in *Arabidopsis*, CPK28 has been identified as a key negative factor dependent in growth stage dependence. CPK28 regulates reactive oxygen-mediated defense signals and can also participate in the tissue-specific balance of JA and Gibberellic Acid (GA; Jin et al., 2017). CPK28 is considered to be a negative regulator of immune signal transduction, and loss of CPK28 function leads to increased resistance to bacterial infection (Monaghan et al., 2014). In vegetative plants, CPK28 phosphorylates and activates two E3 ubiquitin ligases PUB25 and PUB26. These two enzymes target the kinase BIK1, which is required for the activation of PAMP-induced defense signals, for proteasomal degradation (Wang et al., 2018). These two enzymes target the kinase BIK1, which is required for the activation of PAMP-induced defense signals, for proteasomal degradation (Bredow et al., 2021). This negative regulatory mechanism buffered immune signals by controlling BIK1 turnover. In *Nicotiana attenuata*, when *NaCDPK4* and *NaCDPK5* genes, which are homologous to CPK28, were silenced, plants accumulated large amounts of defensive metabolites and showed enhanced resistance to insect predation after injuring or simulating herbivore (Yang et al., 2012). 42 non-redundant CPK subtypes were identified in maize. ZmCPK32 is involved in regulating pollen germination and tube extension (Li et al., 2018). Genome-wide analysis of rice identified 29 CDPKs. OsCPK9 plays an important role in signal transduction of rice blast response (Asano et al., 2005). Plasma membrane-localized GROUP IV CDPKs OsCPK4 and OsCPK18, homologous to CPK28, negatively regulated immunity and enhanced immune signaling and resistance to pathogen infection in functional deficient rice plants (Monaghan, 2018). 24 CDPKs were identified in *Medicago truncatula* (Zhao et al., 2021), CDPK1 silencing in *M. truncatula* altered the expression of cell wall and defense-related genes, resulting in a significant reduction in symbiotic colonization of rhizobium and mycorrhizal bacteria (Ivashuta et al., 2005). We hypothesized that CDPK28 homologues are widely used as negative regulators of immunity in plant species.

In this study, 96, 44 and 57 CDPKs were identified from *Gossypium hirsutum*, *Gossypium raimondii* and *Gossypium arboretum*, respectively. Phylogenetic analysis showed that these CDPKs could be divided into four branches. We further analyzed the gene structure, protein conserved motifs, chromosomal localization and cis-acting regulatory elements of 96 CDPKs in *G. hirsutum*, and analyzed the transcriptome data of upland cotton under stress. Finally, GhCDPK28 (Gh_A11G186100) was screened in upland cotton roots under *V. dahliae* challenge by phosphoproteomics analysis. When the *GhCDPK28-6* gene was silenced in cotton, ROS, lignin and callose accumulation increased, and plant resistance increased. Overexpression of *GhCDPK28-6* in *Arabidopsis* reduces its resistance to disease. Subcellular localization indicated that GhCDPK28-6 was localized in the cell membrane. We also found two proteins, GhPBL9 and GhRPL12C, that may interact with GhCDPK28-6, which provides an idea for further research.

## Materials and Methods

### Plant Materials, Fungal Strain and Growth Conditions

The upland cotton (*G. hirsutum*) cultivar Zhongzhimian2 is a high resistance to *V. dahliae*. After sowing, it was grown in a greenhouse at 23°C (dark)/28°C (light) with a 16 h light/8 h dark photoperiod. The cultivation method of *Nicotiana benthamiana* is the same as the above method. *A. thaliana* (Columbia) was grown in a conditioned greenhouse with a 16 h/8 h photoperiod at 22°C. The relative humidity is 60%.

*Verticillium dahliae* strain Vd080 was used in this study. Mycelium was collected from PDA medium (potato dextrose agar) and cultured in liquid Czapek medium at 120 ~ 140 rpm at 25°C for about 5 days. The concentration was adjusted to 10^6^–10^7^ spores/ml.

### Cloning of *GhCDPK28-6*

Coding sequence (CDS) sequence was searched in cotton database^1^ Primer5.0 was used to design the primer for *GhCDPK28-6* (Supplementary Tabel S1), and the CDS was amplified by high fidelity enzyme. The amplified product was cloned into the pEASY^®^-Blunt Cloning Kit vector, and confirmed by sequencing.

### Sequence Retrieval and Identification of Cotton CDPK Genes

A total of 34 *Arabidopsis CDPK* gene sequences were downloaded from TAIR 10.^2^ The 34 *Arabidopsis* CDPK protein sequences were used as queries to conduct a homologous blast search against *G. hirsutum* (CRI), *G. raimondii* (JGI) and *G. arboretum* (CRI) protein databases.^3^ The molecular weights (kDa) and isoelectric points (pI) of CDPK proteins were determined using ProtParam.

### Phylogenetic, Gene Structure, Conserved Domain Analysis and Chromosomal Mapping

ClustalX 2.0 was used to compare the amino acid sequences of CDPKs identified in cotton and *Arabidopsis*. After conducting a model test, MEGAX software was used to construct the maximum likelihood (ML) phylogenetic tree with the best substitution model (Chang et al., 2020).

Genetic structure shows the server program,^4^ according to the total length of the genome sequence and the corresponding CDS, mapped the structure of the gene exons and introns (Hu et al., 2015). The MEME program^5^ was used to identify the conserved motifs in protein with the default parameters. The conserved domains of GhCDPKs proteins were identified by Batch Web CD-Search Tool^6^ (Lu et al., 2020).

Chromosomal position information about GhCDPKs was comes from annotation files downloaded from the CottonFGD website. Genome collinearity and tandem repeats were detected by MCScanX and CIRCOS with default parameters (Krzywinski et al., 2009).

### Transcriptome Analysis of GhCDPK Responded to Stress

The expression patterns of *GhCDPK* genes under abiotic stress were exhibited through the reads per kb per million reads (RPKM) values from the TM-1 transcriptome data (Accession codes, SRA: PRJNA490626, https://www.ncbi.nlm.nih.gov/bioproject/?term=PRJNA490626; Hu et al., 2019). The expression data of *GhCDPK* genes under *V. dahliae* from KV-1 (Accession codes, SRA: PRJNA89721, https://www.ncbi.nlm.nih.gov/bioproject/PRJNA89721; Sun et al., 2013).

### *GhCDPK28-6* Genes Silenced and Fungal Pathogen Inoculation

*GhCDPK28-6* expression was inhibited by VIGS (Gao et al., 2011). *Agrobacterium tumefaciens* (GV3101) containing *pYL-192* was mixed 1:1 with *A. tumefaciens* containing *pYL- 156- GhCDPK28-6* or *pYL- 156-GhPDS*, *pYL- 156*. *A. tumefaciens* were injected into two fully expanded cotyledons in the same manner as described previously (Zhou et al., 2021). The albino phenotype of *GhPDS* silenced cotton plants was used as the positive control. The expression of *TRV::00* or *TRV:: GhCDPK28-6* plants was detected by RT-qPCR to detect the silencing efficiency. After culture for a week, the plants were inoculated with *V. dahliae*.

### Analysis of ROS, Callose, and the Xylem

3,3′-diaminobenzidine (DAB) staining was used to detection of the production and accumulation of reactive oxygen species in cotton leaves. 12 h after inoculation, leaves of each group of plants were randomly selected and stained with DAB staining solution for 8 h in dark at room temperature. The leaves were placed in 95% ethanol and heated with boiling water until the chlorophyll was completely removed, then soaked in 70% glycerin for microscopic observation and photography. Each experiment was repeated three times.

Phloroglucinol staining was used to observe cotton xylem discoloration. After 24 h of inoculation, five stems of *TRV::00* and *TRV:: GhCDPK28-6* plants were randomly selected and sliced from the same parts, stained with 10% phloroglucinol solution (dissolved in 100% ethanol) for 2 min, incubated in concentrated sulfuric acid for a moment, observed quickly under a microscope and photographed. Three independent biological and technical repeats were performed.

At 48 h after Vd080 inoculation, the leaves of each group were randomly selected to measure callose accumulation. Remove chlorophyll from leaves with a 3:1 volume ratio of ethanol and acetic acid solution, after 3 h of treatment, the leaves were soaked in 70% ethanol and 50% ethanol for 3 h, respectively, and the leaves were soaked in distilled water overnight. Treat with 10% sodium hydroxide for 1 to 2 h, rinse gently, then soak in 0.01% aniline blue solution and incubate in darkness for 3 h. Callose content was observed under the fluorescence microscope by ultraviolet excitation light.

The stems of eight *TRV::00* and *TRV:: GhCDPK28-6* plants were randomly collected at 25 days after inoculation. The same part of the stem was soaked for 40 s in 75% alcohol, and then soaked for 3 min in 3% sodium hypochlorite solution in the clean bench. Finally, the stems were washed with ultrapure water for three times. Stem segments were placed on PDA medium and cultured at 25°C for 7 days.

### Measurements of H_2_O_2_ and NO

Three *TRV::00* or *TRV:: GhCDPK28-6* plants were randomly selected at each time point after Vd080 inoculation. Hydrogen peroxide (H_2_O_2_) and NO was determined using a Quantitative Assay Kit (Jiancheng, Beijing, China). Three independent biological and technical repeats were performed.

### RNA/DNA Extracted and Real-Time Quantitative PCR Analysis

To monitor the expression levels of related resistance genes, leaves of cotton plants with *TRV::00* and *TRV:: GhCDPK28-6* were randomly collected at 0, 1, 3, 6, 9, 12 and 24 h after inoculation. Total RNA was extracted from the collected samples using the RNAprep Pure Plant Kit (TIANGEN, Beijing, China) was used to extract total RNA from leaves. The cDNA was synthesized by using the All-in-One First-Strand cDNA Synthesis Super Mix for qPCR Kit (One-Step gDNA Removal; TransGen, Beijing, China) according to specifications. RT-qPCR was carried out with HiScript^®^ II Q RT SuperMix (Vazyme, Nanjing, China), and the circulate and react according to the instructions. The Roche Light Cycler 480 System (Roche, Mannheim, Germany) was used. The 2^−ΔΔCt^ method was used to calculate the relative fold changes of target genes to analyze the relative expression of cotton defense-related genes. The primers used are listed in Supplementary Table S1. Technical replicates of three independent biological samples were performed.

### Construction and Screening of Transgenic GhCDPK28-6 *Arabidopsis*

The ORF of *GhCDPK28-6* was inserted into the plant expression vector *pCAMBIA2300* and transformed into *Arabidopsis* by floral-dip method (WT; Clough and Bent, 1998). The transformants were screened by 0.1% kanamycin, and the T3 lines with the transgene were identified by PCR and RT-qPCR analysis.

### Plant Disease Resistance Assess

When the leaves of the plant have withered and turned yellow, the diseased plants are divided into 0–4 levels according to the disease severity of the seedlings, and the disease index is calculated according to the following formula:


$$\mathrm{Disease index}=\left[\begin{array}{l} \left(\sum \mathrm{level}\;n\times \mathrm{number of diseased plants}\;\mathrm{at}\;\mathrm{level}\;n\right)/\\ \left(\mathrm{total checked plants}\times 4\right) \end{array}\right]\times 100.$$

All experiments were repeated three times, and more than 80 plants were counted each time (Zhu et al., 2021).

### Subcellular Localization of GhCDPK28-6

*Agrobacterium tumefaciens* containing the vectors (35S-GFP and 35S-GhCDPK28-6-GFP, 35S-GhCDPK28-6^S13F^-GFP, 35S-GhCDPK28-6^S14F^-GFP, 35S-GhCDPK28-6^S15F^-GFP) were cultured to OD600 = 1.0. Resuspend was diluted to OD600 = 0.8 and placed at room temperature for 2 h. Injection was given to the back of the tobacco leaf at 4 weeks of age. After cultured in dark for 24 h, observed by laser scanning confocal microscope (Olympus FV1200). To further verify the subcellular localization of GhCDPK28-6, *35S-GFP* and *35S-GhCDPK28-6-GF*P were transformed into onion epidermal cells by particle bombardment using the PDS-1000/He system (Bio-Rad, USA). Cultivate in MS medium for about 24 h and observe under a confocal microscope. 20% sucrose is used for the plasmolysis experiment (Feng et al., 2021).

### Yeast Two Hybrid Assay

The CDS of *GhCDPK28-6* was cloned into *pGBKT7* vector using as bait vector to screen interacting protein from cDNA libraries in yeast, which is from *G. hirsutum* roots inoculated with *V. dahliae*. The screening interacting protein was sequenced, and further verified the interaction of *pGBKT7-GhCDPK28-6* and *pGADT7-GhPBL9*, *pGADT7-GhRPL12C* by co-transferring into yeast receptive cells (Chang et al., 2020).

### Luciferase Complementation Imaging Assay

As previously described (Chen et al., 2008), *A. tumefaciens* containing the vector (*CAMBIA1300-nLUC* or *pCAMBIA1-cLUC*, *GhCDPK28-6-cLUC*, *GhPBL9-nLUC*, *GhRPL12C-nLUC*) were injected into the tobacco leaf at 4 weeks of age. After 3 days, fluorescence signals were detected using the low-light cooled charge-coupled device camera (Nightshade LB985, BERTHOLDTECHNOLOGIES, Germany).

## Results

### Identification of CDPK Gene Family in Cotton

To identify putative *CDPK* family genes in cotton, we used 34 CDPK protein sequences from *A. thaliana* to perform a homologous blast search on the protein database of *G. hirsutum* (CRI), *G. raimondii* (JGI) and *G. arboretum* (CRI) protein databases.^7^ After that, according to the existence of the conserved CDPK motif identified by the InterProScandatabases,^8^ all candidate GhCDPK proteins selected in these steps are further selected. We obtained 96, 44 and 57 CDPKs in *G. hirsutum*, *G. raimondii* and *G. arboretum*, respectively. The lengths of CDPK proteins ranged from 64 to 907 amino acids, the molecular weight (MW) ranged from 6.726 kDa to 101.033 kDa, and the isoelectric point (IP) ranged from 4.128 to 10.721 (Supplementary Table S2).

### Phylogenetic Analysis of CDPK Gene Family in Cotton

**Figure 1:**
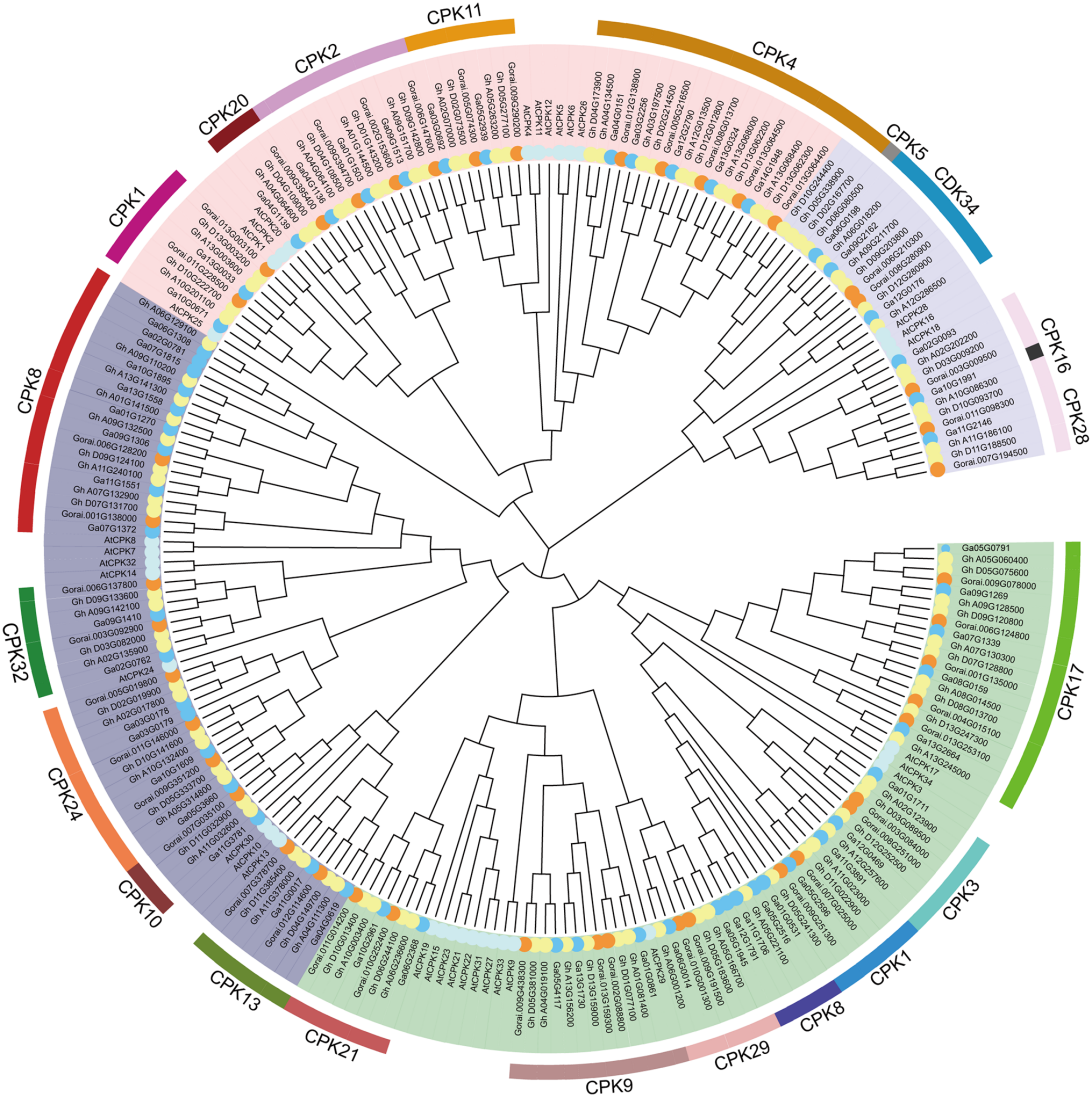
Phylogenetic tree analysis phylogenetic tree. MEGAX software was used to construct the ML phylogenetic tree with the best substitution model. The Protein sequences of *Arabidopsis thaliana* (At), *Gossypium hirsutum* (Gh), *Gossypium raimondii* (Gr) and *Gossypium arboretum* (Gorai) were used. They are divided into four clades, clades I, II, III and VI are represented by green, deep purple, pink and light purple, respectively.

In order to further study the phylogenetic relationship of CDPKs in different cotton varieties. Phylogenetic trees were constructed by ML method using MEGA X software using 96 CDPKs from *G. hirsutum*, 57 CDPKs from *Gossypium arboreum*, 44 CDPKs from *G. raimondii*, and 34 CDPKs from *Arabidopsis* (Figure 1). The results showed that 197 CDPKs were divided into 4 different groups. Among them, group I was the largest with 66 CDPK proteins. Group IV was the smallest with only 26 CDPK proteins. Groups II and III contained 53, 52 CDPK proteins, respectively. In each clade, CDPK members of cotton showed a high degree of similarity to the homologous genes of Arabidopsis. The results showed that CDPKs of these three cotton species were highly conserved.

### Gene Structure, Conserved Motifs and Domain of GhCDPKs

**Figure 2:**
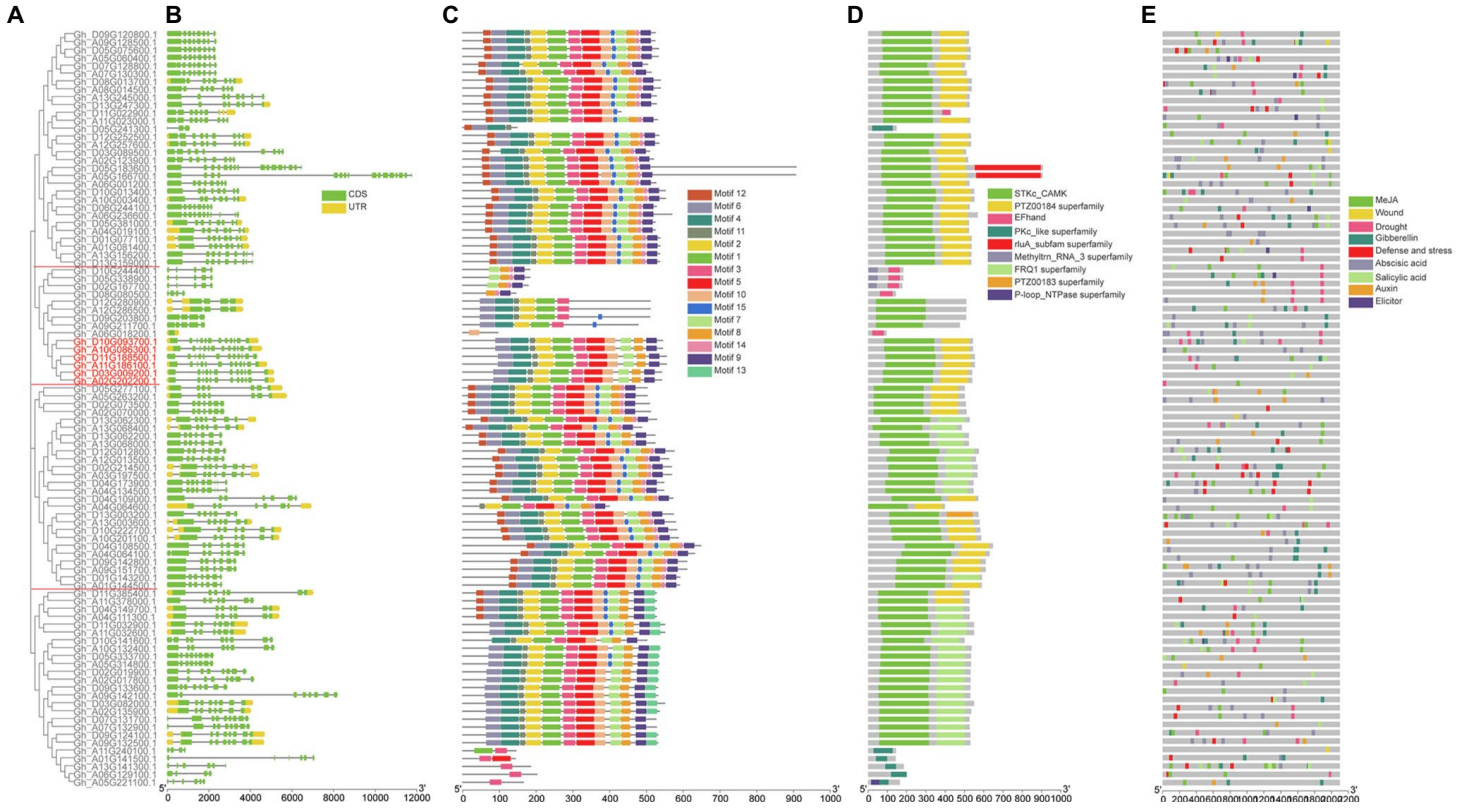
Structural and motif analysis of cotton GhCDPK. **(A)** Phylogenetic relationships between GhCDPKs. The rootless phylogenetic tree was constructed by MEGAX with 1,000 replicates. GhCDPKs are divided into four clades. **(B)** Exon-intron structures of GhCDPK genes. The green box represents coding sequence (CDS), the yellow boxes represent Untranslated Regions (UTR), and the black lines represent introns. **(C)** Conserved motifs of GhCDPK proteins. There are a total of 15 conservative motifs in GhCDPKs, which are represented by different colors. The scale at the bottom shows the length of the protein. **(D)** conserved domain of GhCDPK protein. Nine different conserved domains were identified and indicated in different colours. **(E)** Cis-acting element prediction of GhCDPK genes. Online database PlantCARE was used to analyze cis-acting regulatory elements in the 2 kb promoter region upstream of all GhCDPKs. Cis-acting regulatory elements related to MeJA, wounding, drought, GA, defense, abscisic acid, salicylic acid, auxin and elicitor were predicted.

We constructed a separate unrooted phylogenetic tree based on DNA sequence, and compared and analyzed the exon-intron structure to further understand the phylogenetic relationship and gene structure of the *G. hirsutum* CDPK family. The results showed that cotton CDPK protein was divided into four subgroups (Figure 2A). *GhCDPK* gene sequences were significantly different in length, ranging from 2 to 12 kb, *GhCDPK* genes possessed at least 1 exon and most 16 exons (Figure 2B). The intron distribution of *GhCDPK* gene family is abundant, and the genes with similar sequence have similar intron distribution. This suggests that the exon-intron structure is highly correlated with the phylogenetic relationship between *GhCDPK* genes.

**Figure 3:**
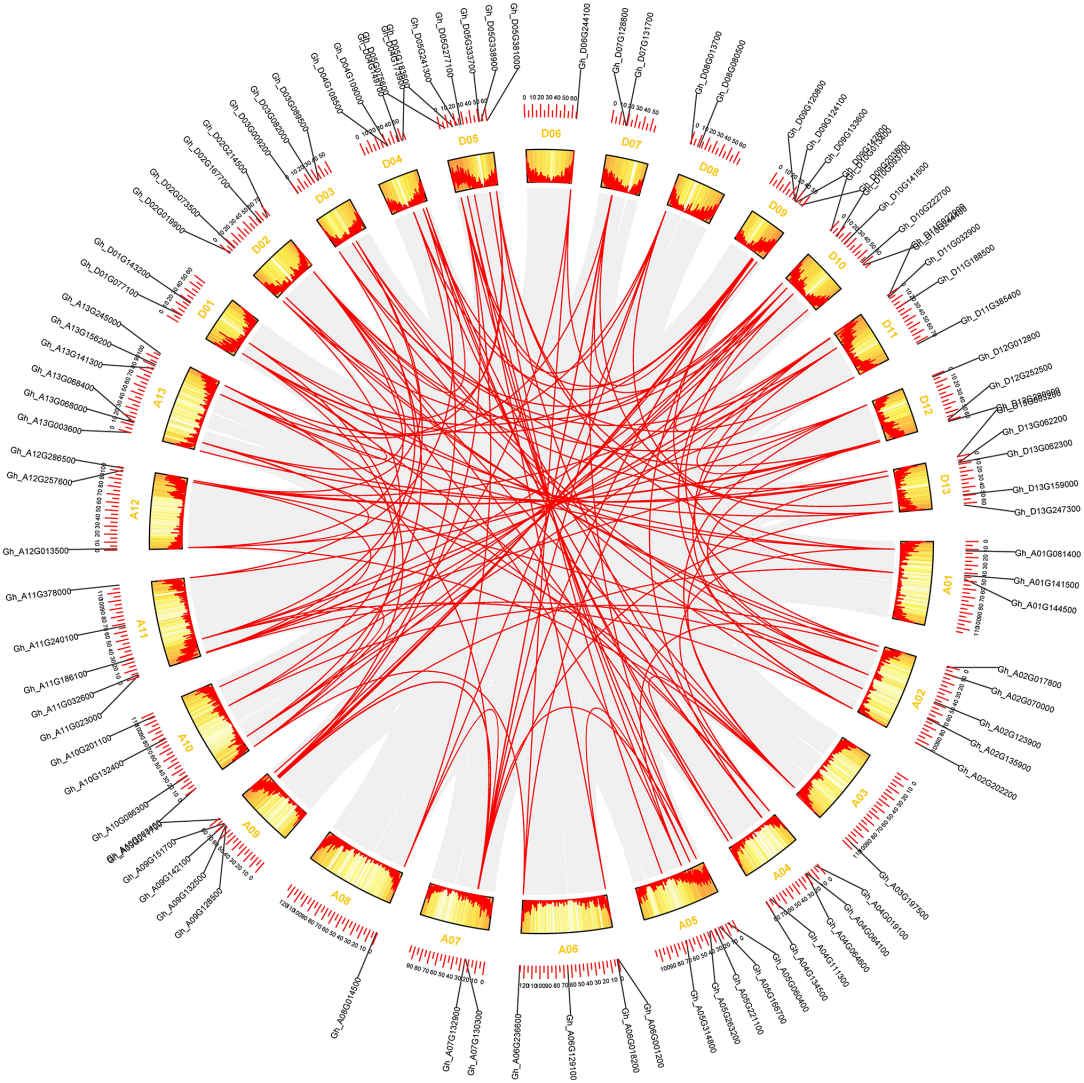
The synteny relationship of GhCDPK genes. MCScanX was used to detect genome collinearity and tandem repeats under default parameters, and CIRCOS was used to display collinearity. The red line represents paralogous gene pairs.

The conserved motifs of GhCDPK protein were identified by MEME software. Fifteen putative motifs named motifs 1–15, were finally identified. Most GhCDPK proteins have 11 conserved motifs, and their numbers are 1–11, indicating that these motifs are highly conserved in GhCDPK (Figure 2C). In addition, we also identified domains in the GhCDPK using Batch Web CD-Search Tool, closely related proteins had more similar arrangements of motifs and domains (Figure 2D).

The six GhCDPK28 marked in red have similar gene sequence length and intron distribution, all have Motif 1–11, and all have STKc-CAMK and PTZ00184 superfamily domins (Figures 2A–D).

### Prediction of Cis-Acting Elements in the Promoters of GhCDPKs

To better understand the possible biological functions of GhCDPKs, we analyzed cis-acting regulatory elements in the upstream 2 kb promoter region of all GhCDPKs using the online database PlantCARE. We found that the predicted cis-acting elements were related to transcription, hormones, stress response, cell cycle, and development. Response to various stresses were the main focus, for instance methyl jasmonate (MeJA), wounding, drought, GA, defense, abscisic acid, SA, auxin, elicitor etc. (Figure 2E). The abundance of cis-acting elements suggests that *GhCDPK* may have a variety of biological functions in upland cotton.

GhCDPK28 contains cis-acting elements associated with drought, Abscisic acid, MeJA, GA, SA and auxin. Among them, Gh_D10G093700.1 contains cis-acting elements associated with drought, GA, abscisic acid and SA. Gh_A10G086300.1 contains cis-acting elements associated with MeJA, abscisic acid and auxin. Gh_D11G188500.1 contains cis-acting elements associated with drought, abscisic acid, MeJA, GA and SA. Gh_A11G186100.1 contains cis-acting elements associated with drought, MeJA and SA. Gh_D03G0092001.1 Contains SA, auxin associated Cis-acting contains cis-acting elements associated with SA and auxin. Gh_A02G2022001.1 contains cis-acting elements associated with drought and MeJA (Figure 2E).

### Chromosomal Location and Gene Synteny Analysis of GhCDPKs

The approximate location analysis of *GhCDPK* gene on cotton chromosome showed that *GhCDPK* distributed on 26 chromosomes of D and A subgenome (Figure 3; Supplementary Figure S2). In detail, chromosomes D05 contained seven *GhCDPK* genes, A13 contained six *GhCDPK* genes, A02, A04, A05, A09, A11, D09 D10 and D13 contained five *GhCDPK* genes, A06, A10, D02, D04 and D11 contained four *GhCDPK* genes, A01, A12, D03 and D12 contained three *GhCDPK* genes, A07, D01, D07 and D08 contained two *GhCDPK* genes, and A03, A08 and D06 only contained one *GhCDPK* gene.

### Transcriptome Analysis

**Figure 4:**
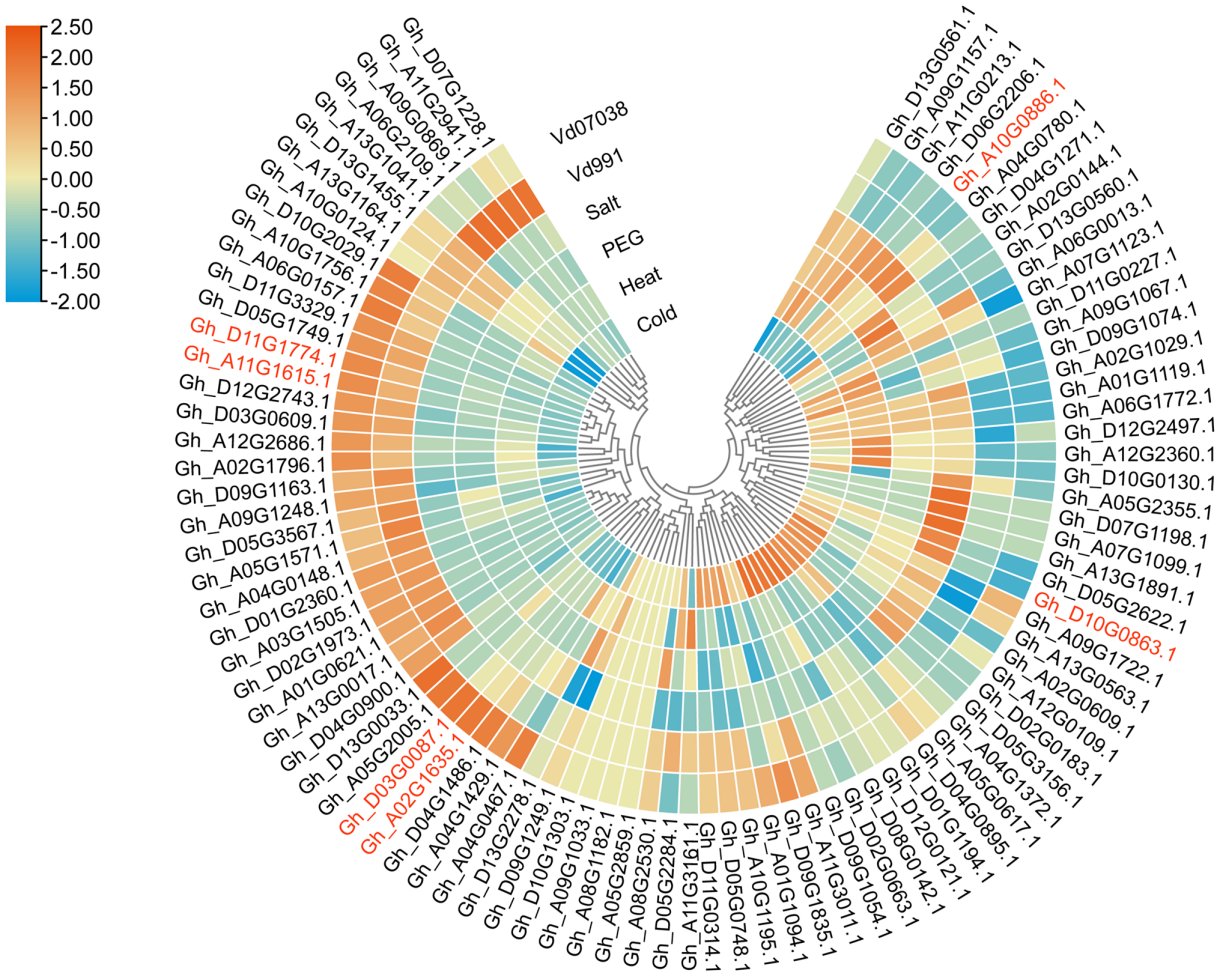
Expression pattern analysis of *GhCDPK* genes under stress. The expression patterns of *GhCDPK* genes under abiotic stress were exhibited through the reads per kb per million reads (RPKM) values from the TM-1 transcriptome data. The expression datas of GhCDPK genes under *Verticillium dahliae* from KV-1.

To determine which *GhCDPK* genes potentially function in defense and stress, transcriptome data was used to study the expression patterns of members of upland cotton under pathogen, salt, drought, cold and heat stress (Figure 4). The majority of *GhCDPK* genes from the same subfamily had similar expression patterns. After receiving Vd07038 stress, 37 *GhCDPK* genes expressions were up-regulated. After receiving Vd991 stress, 36 *GhCDPK* genes expressions were up-regulated. Interestingly, these genes that were up-regulated in pathogen stress (*V. dahliae* Vd991 and Vd07038) were down-regulated in abiotic stress (salt, drought, cold and heat). These results indicate that GhCDPKs have different roles in abiotic and biotic stress responses of cotton.

The six GhCDPK28 have different expression patterns under biotic or abiotic stress. Gh_D10G0863.1 (IDs in major assembly is Gh_D10G093700.1) and Gh_A10G0886.1 (Gh_A10G086300.1) was up-regulated under salt stress, and down-regulated under Vd0738 and Vd991 stress. Gh_D03G0087.1 (Gh_D03G009200.1) was up-regulated under Vd0738, but there was no significant change under Vd991 stress, and down-regulated under abiotic stress. Gh_D11G1774.1 (Gh_D11G188500.1) and Gh_A11G1615.1 (Gh_A11G186100.1) was up-regulated under Vd0738 and Vd991 stress, and down-regulated under abiotic stress, which attracted our attention. Early proteomic analysis showed that a certain peptide was phosphorylated after inoculation of *V. dahliae*, and amino acid sequence analysis revealed that the peptide was Gh_A11G186100.1 after BLAST (Supplementary Figure S1). Therefore, we further explored whether Gh_A11G186100.1 (named as GhCDPK28-6) plays a role in cotton resistance to Verticillium wilt.

### Silences Enhanced the Resistance of Cotton to *V. dahliae*

**Figure 5:**
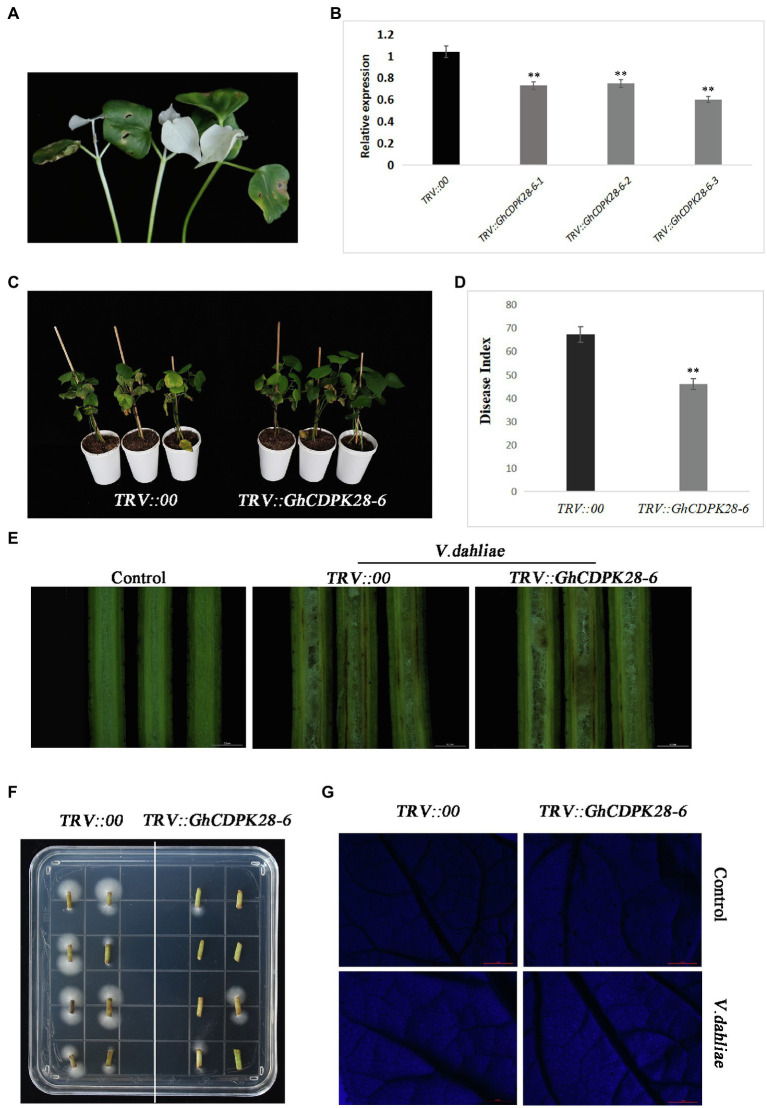
Knock-down of *GhCDPK28-6* enhances plant resistance to *V. dahliae*. **(A)** *TRV::GhPDS* plants showed albino phenotype as positive control. **(B)** Expression of *GhCDPK28-6* gene in plants. **(C)** pathogenetic phenotypes of control and *GhCDPK28-6*-silenced plants; **(D)** Disease symptoms of plants at 25 dpi after inoculation. **(E)** Stem vascular bundle browning. The scale is 0.2 cm. **(F)** *V. dahliae* recovery assay. **(G)** Callose deposition in cotton leaves, the scale is 5 mm. The error bar represents standard deviation of three biological replicates. Asterisks indicate statistically significant differences (^**^p < 0.001, Student’s *t*-test).

We used VIGS technology to verify whether GhCDPK28-6 plays a role in cotton resistance to *V. dahliae*. The approximately 250 bp GhCDPK28-6 CDS was integrated into the vector pTRV2 to specifically silence the expression of GhCDPK28-6 gene. When the albino phenotype appeared in *TRV::GhPDS* infected newly true leaves (Figure 5A), RT-qPCR was used to detect gene silencing efficiency of *TRV:00* and *TRV:GhCDPK28-6* plants. The results showed that *TRV::GhCDPK28-6* gene silencing was successful (Figure 5B). After inoculated with pathogen Vd080 by root dipping method, the wilting and chlorosis changes of leaves of *TRV::00* plants were more serious than those of (Figures 5C,D), and the brown changes of vascular bundles were more obvious (Figure 5E). The disease index of *TRV::00* and *TRV::GhCDPK28-6* plants were 67.47 and 44.65, respectively (Figure 5D). Fungal recovery was measured in stem segments of inoculated cotton plants. As shown in the figure, the *TRV::GhCDPK28-6* plants fungi colonized faster (Figure 5F). After infection with Vd080, Callose deposits were more dense (number per cm^2^) in true leaves of *TRV::GhCDPK28-6* plants than in *TRV:00* plants (Figure 5G). The above results indicate that the silencing of *GhCDPK28-6* improves the resistance of cotton to *V. dahliae*, indicating that *GhCDPK28-6* may be a negative regulator in the resistance of plants to pathogen infection.

### Expression Levels of Disease-Resistant Genes

**Figure 6:**
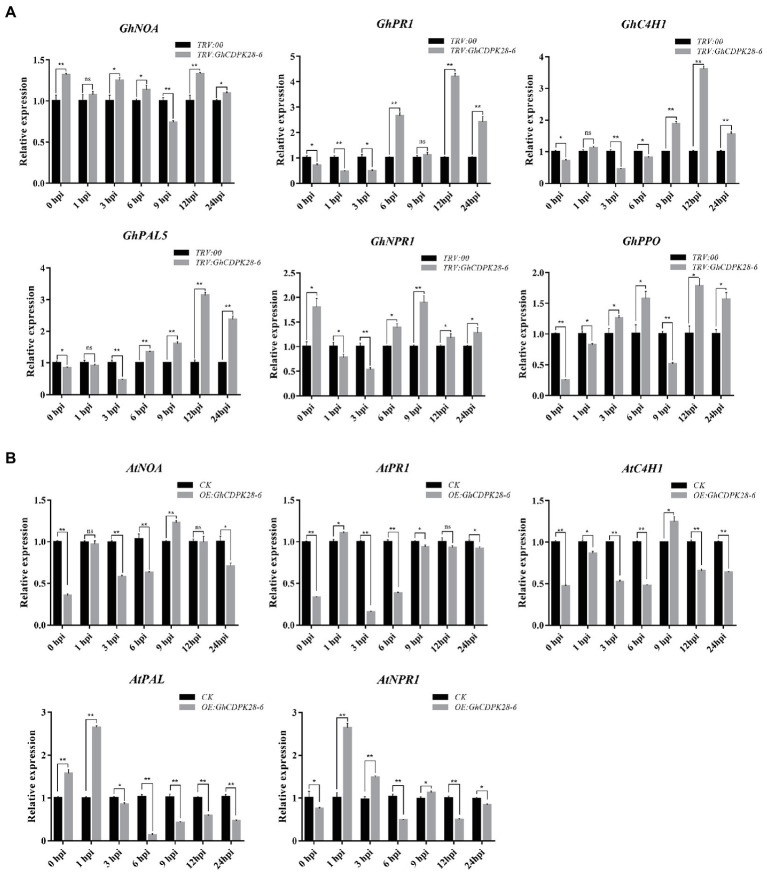
Expression of disease-resistance related genes in silenced cotton and transgenic *Arabidopsis*. **(A)** RT-qPCR analysis of six disease-resistance related genes in leaves of cotton. The *GhUBQ7* gene was used as the reference gene. **(B)** RT-qPCR analysis of five disease-resistance related genes in leaves of *Arabidopsis*. *AtUBQ10* as the internal control gene. The error bar represents standard deviation of three biological replicates. Asterisks indicate statistically significant differences (^*^p < 0.05; ^**^p < 0.001, Student’s *t*-test).

To investigate whether *GhCDPK28-6* affects plant disease resistance at the transcriptional level, we detected the expression of plant disease-resistance related genes by RT-qPCR. In cotton, the expression level of these genes in *TRV:: GhCDPK28-6* plants was generally higher than that in *TRV:: 00* plants (Figure 6A). In *TRV:: GhCDPK28-6* plants, *GhNOA* expression was higher at 0, 3, 6, 12 and 24 hpi; *GhPR1* expression was higher at 6, 12 and 24 hpi, *GhC4H1* expression was higher at 9, 12 and 24 hpi; *GhPAL* expression was higher at 6, 9, 12 and 24 hpi; *GhPPO* expression was higher at 3, 6, 12 and 24 hpi; GhNPR1 expression was higher 0, 6, 9, 12 and 24 hpi.

### GhCDPK28-6 Is Involved in Plant Resistance to *V. dahliae* by Regulating Reactive Oxygen Species and Lignin Levels

**Figure 7:**
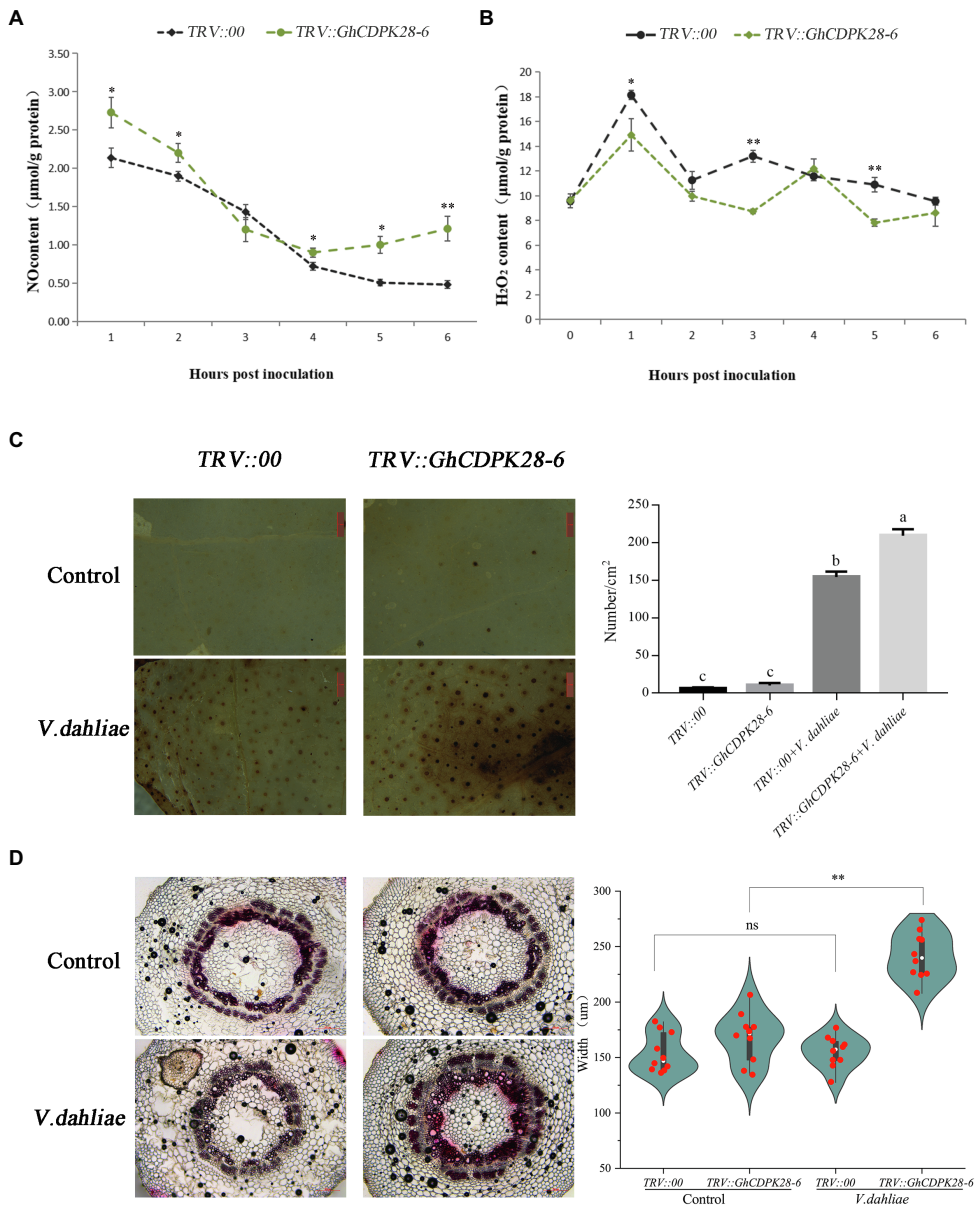
Accumulation of NO and H_2_O_2_, phenotypes of reactive oxygen species bursts and lignin deposition in *GhCDPK28-6* silenced and control plants. **(A)** NO content in roots of silenced plants and control plants within 6 h after *V. dahliae* inoculation. **(B)** H_2_O_2_ content in roots of silenced plants and control plants within 6 h after *V. dahliae* inoculation. **(C)** Production and accumulation of ROS in cotton leaves after 12 h of *V. dahliae* inoculation, and quantitative analysis of ROS. DAB staining was used for detection. It was observed under the stereomicroscope with 10× amplification and the number of stained spots per cm^2^ was recorded. The scale is 1 mm. **(D)** After 72 h of inoculation, cotton vascular bundles were stained with phloroglucinol and their widths were recorded. The scale is 200 μm. The experiment was repeated three times (^*^p < 0.05; ^**^p < 0.001, Student’s *t*-test).

To explore how GhCDPK28-6 plays a role in plant disease resistance, we measured the content of reactive oxygen species (ROS) and xylem accumulation in plants. ROS burst are one of the indicators for evaluating plant disease resistance. At 0, 1, 3 and 6 h after Vd080 infection, NO content in roots of *TRV:: GhCDPK28-6* plants was higher than that of *TRV::00* plants, but H_2_O_2_ content was lower than that of *TRV::00* plants (Figures 7A,B). Moreover, DAB staining results showed that compared with the control plants, the leaves of the silenced plants were stained more darkly and the stained area was larger, indicating that the ROS level in the leaves of the *TRV:: GhCDPK28-6* plants was higher than that of the *TRV::00* plants 12 h after inoculation (Figure 7C). Phloroglucinol staining showed that the stained area of xylem in the stems of plants infected by Vd080 was larger than that of uninfected plants. When infected by pathogenic bacteria, the stained area of xylem of *TRV:: GhCDPK28-6* plants is larger than that of *TRV::00* plants (Figure 7D). These results suggest that *GhCDPK28-6* may be participate in cotton resistance to Vd080 by regulating ROS and lignin content.

### Overexpression of *GhCDPK28-6* in *Arabidopsis* Reduces Plant Resistance

**Figure 8:**
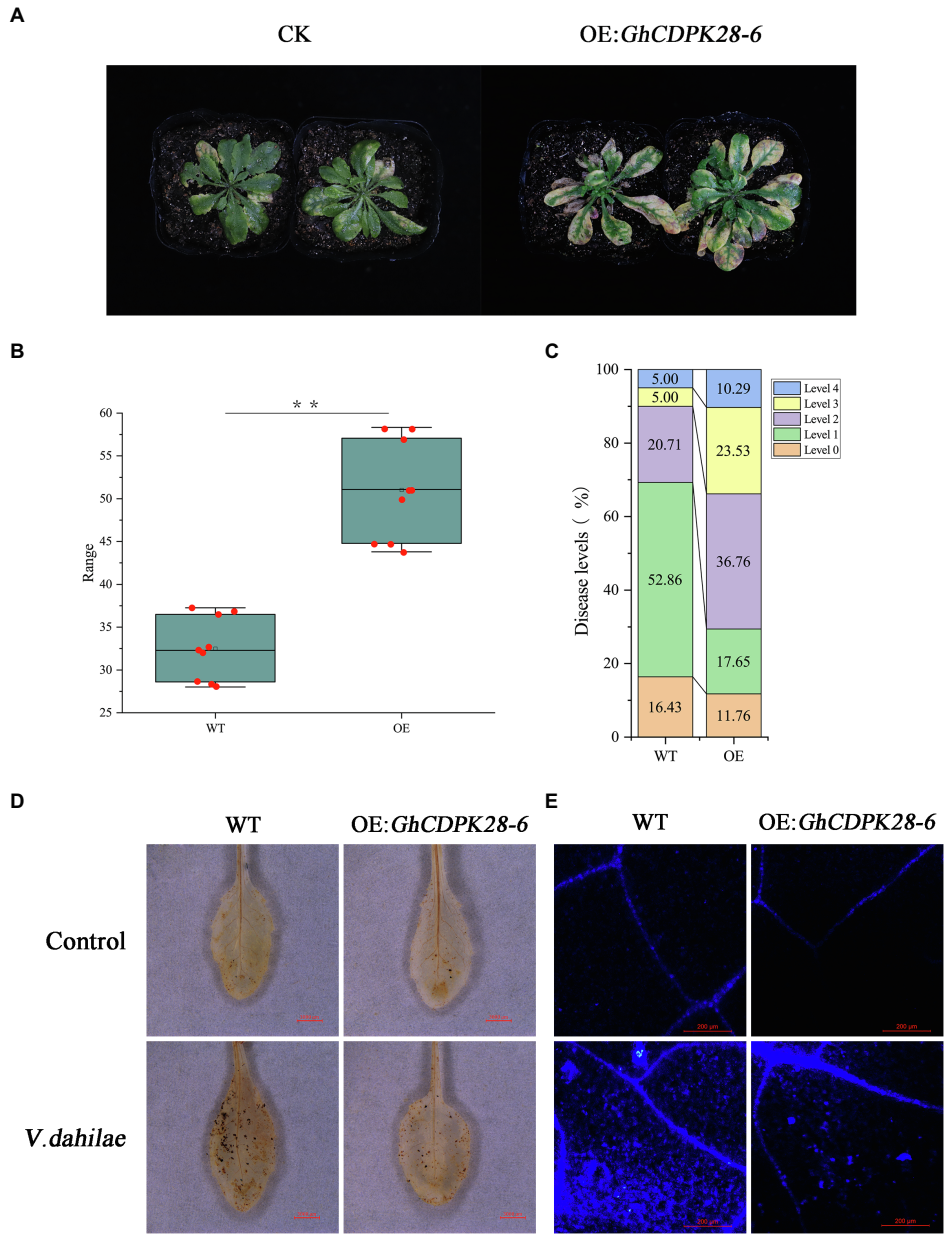
Overexpression of *GhCDPK28-6* in *A. thaliana* attenuated its resistance to *V. dahliae*. **(A)** Phenotype of *A. thaliana* after vd080 inoculation in soil. **(B,C)** Disease index. **(D)** DAB staining was used to detect the production and accumulation of ROS in *A. thaliana* leaves at 12 h after inoculation. It was observed under the stereomicroscope with 10× amplification. The scale is 3,000 μm. **(E)** Callose deposition in cotton leaves, the scale is 200 μm. The error bar represents standard deviation of three biological replicates. Asterisks indicate statistically significant differences (^**^p < 0.001, Student’s *t*-test).

The *pCAMBIA2300* vector was used to transfer *GhCDPK28-6* into wild-type *Arabidopsis*. The homozygous transgenic lines overexpressing *GhCDPK28-6* were screened and confirmed by 0.1% kanamycin, PCR and qPCR (Supplementary Figure S3), and finally four lines were determined for the next experiment. The spore suspension was inoculated with T3 generation *Arabidopsis* and WT. The results showed that the resistance of transgenic plants (OE: *GhCDPK28-6*) to *V. dahliae* was weakened, and the disease index of wild-type and transgenic plants were 38.75 and 57.5, respectively (Figures 8A–C). After pathogen inoculation, the ROS level in the leaves of the WT plants was higher than that of the transgenic plants (Figure 8D), the accumulation of callose in the WT plants was higher than that in the transgenic plants (Figure 8E). Since *Arabidopsis* does not contain polyphenol oxidase (PPO), we examined the expression levels of another five disease-resistance related genes in overexpressed *Arabidopsis* plants. As expected, these disease-resistance related genes were down-regulated at most time points after inoculation in overexpressed *A. thaliana* (Figure 6B). The results showed that *GhCDPK28-6* overexpression plants were more susceptible to bacterial pathogen.

### Subcellular Localization

**Figure 9:**
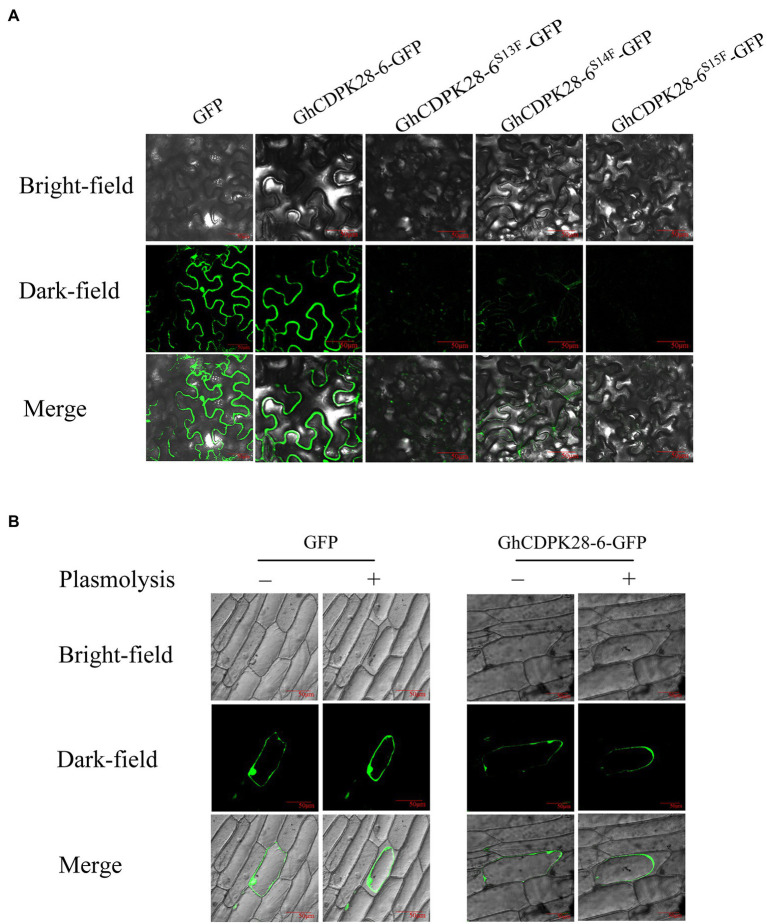
Subcellular localization of GhCDPK28-6, GhCDPK28-6^S13F^, GhCDPK28-6^S14F^and GhCDPK28-6^S15F^. **(A)** Subcellular localization of GhCDPK28-6, GhCDPK28-6^S13F^, GhCDPK28-6^S14F^and GhCDPK28-6^S15F^ in *Nicotiana benthamiana*. **(B)** Subcellular localization of GhCDPK28-6 in onion. In the plasma wall separation experiment, cells were treated with 20% sucrose.

In the early stage, we found phosphorylation of threonine 13, 14 and 15 in GhCDPK28-6 through proteomic analysis of *G. hirsutum* roots infestation by *V. dahliae*. To investigate whether phosphorylation site mutations affect the subcellular localization of GhCDPK28-6, we mutated serine at 13, 14, and 15 to phenylalanine. A *GhCDPK28-6-GFP* vector was constructed and instantly expressed on the back of 4-week-old tobacco leaves by *A. tumefaciens* injection. The results showed that GhCDPK28-6 was located on the cell membrane of tobacco cells (Figure 9A). To further demonstrate the membrane localization of GhCDPK28-6, the localization of GhCDPK28-6 in onion cells was observed by gene gun method. As expected, GhCDPK28 was localized in the cell membrane after separation from the cytoplasmic wall of onion cells (Figure 9B). GssshCDPK28-6^S13F^ and GhCDPK28-6^S15F^ could not be located on the membrane in tobacco, and the membrane localization signal of GhCDPK28-6^S14F^ became very weak (Figure 9A).

### GhCDPK28-6 Interacts With GhPBL9 and GhRPL12C

**Figure 10:**
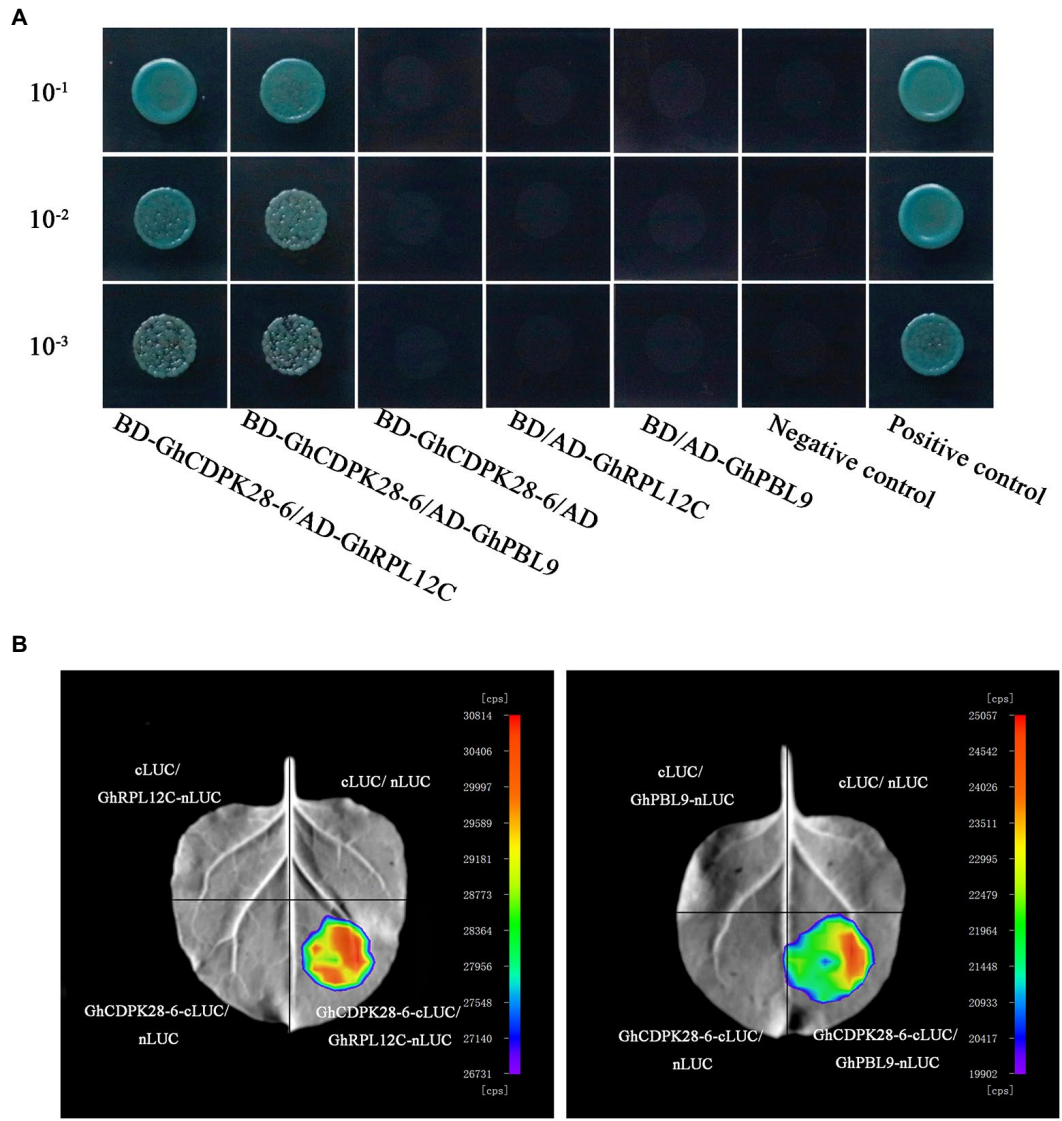
GhCDPK28-6 interacts with GhPBL9 and GhRPL12C. **(A)** Yeast two-hybrid assays of the interactions of GhCDPK28-6 with GhPBL9 and GhRPL12C. Transformants were grown on SD/-Leu/-Trp/-Ade/-His (+ X-α-gal) media. pGBKT7-53/pGADT7-RecT was used as the positive control. pGBKT7-Lam/pGADT7-RecT were used as negative controls. **(B)** LCI was used to detect the interaction of GhCDPK28-6 with GhPBL9 and GhRPL12C in *N. benthamiana* leaves.

To further study the mechanism of GhCDPK28-6 in cotton, two interacting proteins, Probable serine/threonine-protein kinase PBL9 (GhPBL9, Gh_A05G354600) and 60S ribosomal protein L12-3 (GhRPL12c, Gh_A09G169800), were screened from upland cotton roots inoculated with *V. dahliae* by using *pGBKT7- GhCDPK28-6* vector as bait vector. To confirm this interaction, 1:1 Yeast Two Hybrid (Y2H) was performed between GhCDPK28-6 fused with the Gal4 binding domain (*BD- GhCDPK28-6*) and GhPBL9/GhRPL12Cfused to the Gal4 activation domain (*GhPBL9-AD/GhRPL12C-AD*). GhCDPK28-6 was observed to interact with GhPBL9 and GhRPL12C in yeast, respectively (Figure 10A). Luciferase Complementation Imaging (LCI) assays were performed to test the interaction of GhCDPK28-6 with GhPBL9 and GhRPL12C in *N. benthamiana* cells (Figure 10B).

## Discussion

*Verticillium dahliae* is a soil-borne hemibiotrophic fungus which is the most destructive disease in cotton production (Hu et al., 2021). Plants have evolved a sophisticated immune system to fight infection by pathogens (Defalco and Zipfel, 2021). Plant cells that recognize *V. dahliae* microbio-related molecular patterns, or internal effectors, immediately trigger signal transductions that lead to rapid defense responses, including massive transcriptional reprogramming.

Whole genome sequencing of CDPK genes has been widely carried out in some plants to help identify important genes, such as 34 CDPKs in Arabidopsis (Hrabak et al., 2003), 31 CDPKs in rice (Asano et al., 2005), 29 CDPKs in tomato (Wang et al., 2016a), 30 CDPKs in poplar (Zuo et al., 2013). In this work, CDPKs was divided into four groups based on phylogenetic trees of *A. thaliana*, *G. hirsutum*, *G. raimondii* and *G. arboretum* (Figure 1), which were consistent with other plants species (Zhao et al., 2021). Some CDPK genes were only found in *A. thaliana*, but lost in *Gossypium*, suggesting that gene loss has occurred since *A. thaliana* and *Gossypium* diverged from their common ancestor. Members of GhCDPKs from the same group have similar exon-intron structure, conserved motif compositions, conserved domain and Cis-acting element, indicating that they are closely evolutionary conservation (Figure 2). The promoter region of GhCDPK contains cis-acting elements related to transcription, hormones, stress response, cell cycle and development, suggesting that GhCDPKs has a potential role in regulating plant hormone environmental stress, growth and development (Figure 2E). The expression pattern of *GhCDPK* genes was analyzed to understand its potential function. The results showed that GhCDPKs had different functions in abiotic and biological stress responses. The expression of *GhCDPK28* was up-regulated under biotic stress (*V. dahliae* Vd991 and Vd07038) and down-regulated under abiotic stress (salt, PEG, heat and cold; Figure 4).

Calcium-dependent protein kinases are the key proteins of plant signal transduction, which transmit important second messenger Ca^2+^ through phosphorylation of various substrates (Li et al., 2019a). CDPKs play an important role in various physiological reactions, such as stem and root development, pollen tube growth, stomatal movement, hormone signal transduction, transcriptional reprogramming, and stress resistance, etc. AtCPK11 and AtCPK24 jointly mediate Ca^2+^-dependent inhibition of K^+^ in channels, thereby regulating the growth of *Arabidopsis* pollen tubes (Zhao et al., 2013), and AtCPK11 is also involved in root hair elongation (Vijayakumar et al., 2016). AtCPK10/30/32 of subgroup III can respond to nitrate signals and is an important regulator of stem and root development (Liu et al., 2017). CDPKs play an essential role in a plant defense response. Over-expression of CDPK13 confers cold tolerance on rice plants (Komatsu et al., 2007). AtCPK5/6/11 from subgroup I were reported as redundant positive regulators of defense responses downstream of multiple PAMPs/DAMPs (Zhao et al., 2013). Overexpression of AtCPK1 or AtCPK5 can trigger the accumulation of SA, leading to broad-spectrum pathogen resistance (Coca and Segundo, 2010; Dubiella et al., 2013). NtCDPK2 plays a role in gene-for-gene (Avr9-Cf9) fungal resistance in tobacco (Romeis et al., 2001). GhCPK33 negatively regulates defense against *V. dahliae* through phosphorylation of GhOPR3 (Hu et al., 2018). The *Arabidopsis* CDPK CPK28 attenuates the immune response and antimicrobial immunity induced by PAMP and is a negative regulator of immune signal (Monaghan et al., 2014). As shown in the figure, the wilting and yellowing of *TRV::00* were more serious than that of *GhCDPK28-6* in cotton, and vascular bundle browning was significantly changed after inoculation with Vd080 (Figures 5C,E). Determination of fungal recovery in cotton stems showed that the colonization rate of *TRV::00* was significantly higher than that of *TRV:: GhCDPK28-6* (Figure 5F). Overexpression of *GhCDPK28-6* reduced the resistance of transgenic *A. thaliana* to *V. dahliae* (Figure 8). These results indicate that *GhCDPK28-6* played a negative regulatory role in plant resistance to pathogenic fungus infection.

Plants trigger a series of defense responses to fight off fungal infections (Zhu et al., 2021), including the outbreak ROS, xylem thickening callose accumulation, and so on (Yang et al., 2018). ROS is a major immune signaling molecule that also mediates cell wall modification (Oger et al., 2012) and is a key regulator that plays a role in the post-translational modification of defense-related proteins (Xu et al., 2011). The ROS- and NO-mediated signaling pathways interact to help plants cope with biological or abiotic stresses (Kim et al., 2017). In our study, the NO content in *TRV:: GhCDPK28-6* plants was significantly higher than that in *TRV::00* plants at 0, 1, 3, 6 hpi (Figure 7A). H_2_O_2_ content in *TRV:: GhCDPK28-6* plants was lower than that in *TRV::00* plants at 1, 3, 6 hpi, and tended to be similar at 12 hpi (Figure 7B). However, DAB staining at 12 h after inoculation showed that the staining area of *GhCDPK28-6* silenced plants was larger (Figure 7C). These results showed that ROS content in leaves of *TRV:: GhCDPK28-6* cotton plants decreased first and then increased after inoculation with *V. dahliae*. Callose-containing cell-wall appositions are induced in the early stages of pathogen invasion to form a barrier. It is a marker of the plant’s defense response (Han et al., 2021). The accumulation of lignin in cotton secondary cell wall increased the resistance of the plant to *V. dahliae* infection (Tang et al., 2019). The deposition of callose (Figure 5G) and lignin (Figure 7D) in silencing plants was higher than that in control plants.

Nitric oxide associated factor (NOA) participate in the pathway of NO synthesis and are important genes related to plant disease resistance (Tewari et al., 2019). NO donor or NO synthase can induce expression of pathogenesis-related 1 protein (PR1) and phenylalanine ammonia-lyase (PAL) defense-related genes in plants (Durner et al., 1998). PAL and cinnamate 4-hydroxylase (C4H) are the core enzymes in lignin synthesis and play a role in plant disease resistance by regulating lignin content. Polyphenol oxidase (PPO) plays a pivotal role in plant disease resistance by catalyzing the production of lignin and phenols (Wu et al., 2019). NPR1 is an important gene related to disease resistance in plants, which participate in the process of SA-induced PR gene expression (Ding et al., 2018). These six disease-resistance related genes were up-regulated in *TRV:: GhCDPK28-6* plants (Figure 6A) was consistent with the enhanced ROS burst and increased xylem deposition in silenced plants (Figure 7).

Pattern recognition receptor can interact with BIK1 and PBS1-like (PBL) proteins and activate downstream immune signals (Ranf et al., 2014). In Arabidopsis, AtCPK28 reduces the BIK1-mediated immune response by phosphorylation and disrupting the stability of BIK1. The interaction protein GhPBL9 of GhCDPK28-6 screened by us is homologous to AtBIK1. The other GhCDPK28-6 interaction protein we screened, RPL12C, is a 60s ribosomal protein, evidence shows that phosphorylation of the RPL12 affects translation during mitosis (Imami et al., 2018). RPL12 and RPL19 are involved in resistance to non-host diseases and virulent pathogens (Nagaraj et al., 2016).

Calcium-dependent protein kinase protein has four conserved characteristic domains. Among them, the N-terminal variable sequences potentially important for subcellular localization of CDPKs (Harper et al., 1991). Phosphorylation sites in the N-terminal variable domain may be related to accessibility and specificity of substrates (Matschi et al., 2013). In *A. thaliana*, CPK28 locates on the cell membrane, as do groups IV CDPKs OsCPK4 and OsCPK18, which are homologous to CPK28 (Monaghan, 2018). AtCPK28 loses its original membrane localization after glycine mutation at the second site of the N-terminal (Monaghan et al., 2014). In our study, sequence alignment showed that GhCDPK28-6 and AtCPK28 were homologous, and GhCDPK28-6 subcellular localization was on the cell membrane. However, inactivation mutations at phosphorylation sites 13 and 15 of GhCDPK28-6 alter membrane localization, and mutations at phosphorylation site 14 attenuated membrane localization (Figure 9). At present, it is still unclear whether the phosphorylation of GhCDPK28-6 affects its function, and what are its key phosphorylation sites, which will be the focus of our next research.

Collectively, this study investigated the relationship between GhCDPK28 and resistance to Verticillium wilt in cotton. When the *GhCDPK28-6* gene was silenced in cotton, ROS, lignin and callose accumulation increased, and plant resistance increased. Whereas *GhCDPK28-6* overexpression plants were more susceptible to *V. dahliae*. Subcellular localization indicated that GhCDPK28-6 was localized in the cell membrane. GhCDPK28-6 interacts with GhPBL9 and GhRPL12C. It suggests that GhCDPK28-6 may be a potential molecular target for regulating cotton’s resistance to Verticillium wilt.

## Data Availability Statement

The original contributions presented in the study are included in the article/Supplementary Material, further inquiries can be directed to the corresponding authors.

## Author Contributions

YW, HF, and HZ designed the experiment. YW implemented and collected the data. JZ performed the bioinformatics analysis. YW and JZ analyzed the results and prepared the manuscript. HF, HZ, WH, JZ, LeZ, XZ, ZF, FW, LiZ, and YZ revised the manuscript. All authors revised and approved the final manuscript.

## Funding

This work was supported by the Natural Science Foundation of Henan Province (No. 212300410418), the Central Public Interest Scientific Institution Basal Research Fund (No. 1610162021031) and the Agricultural Science and Technology Innovation Program of Chinese Academy of Agricultural Sciences.

## Conflict of Interest

The authors declare that the research was conducted in the absence of any commercial or financial relationships that could be construed as a potential conflict of interest.

## Publisher’s Note

All claims expressed in this article are solely those of the authors and do not necessarily represent those of their affiliated organizations, or those of the publisher, the editors and the reviewers. Any product that may be evaluated in this article, or claim that may be made by its manufacturer, is not guaranteed or endorsed by the publisher.

## Footnotes

^1^ https://cottonfgd.org/profiles/gene/Gh_A11G1615/

^2^ http://www.arabidopsis.org/

^3^ https://www.cottongen.org

^4^ http://gsds.cbi.pku.edu.cn/

^5^ https://meme-suite.org/meme/tools/meme

^6^ https://www.ncbi.nlm.nih.gov/Structure/bwrpsb/bwrpsb.cgi

^7^ https://cottonfgd.org/sequenceserver/

^8^ https://www.ebi.ac.uk/interpro/search/sequence/

## References

[ref1] Alves H. L. S. Matiolli C. C. Soares R. C. Almadanim M. C. Oliveira M. M. Abreu I. A. (2021). Carbon/nitrogen metabolism and stress response networks - calcium-dependent protein kinases as the missing link? J. Exp. Bot. 72, 4190–4201. doi: 10.1093/jxb/erab136, PMID: 33787877 PMC8162629

[ref2] Asano T. Tanaka N. Yang G. Hayashi N. Komatsu S. (2005). Genome-wide identification of the rice calcium-dependent protein kinase and its closely related kinase gene families: comprehensive analysis of the CDPKs gene family in rice. Plant Cell Physiol. 46, 356–366. doi: 10.1093/pcp/pci035, PMID: 15695435

[ref3] Atallah Z. K. Maruthachalam K. Vallad G. E. Davis R. M. Klosterman S. J. Subbarao K. V. (2011). Analysis of *Verticillium dahliae* suggests a lack of correlation between genotypic diversity and virulence phenotypes. Plant Dis. 95, 1224–1232. doi: 10.1094/PDIS-02-11-0110, PMID: 30731695

[ref4] Boudsocq M. Droillard M. J. Regad L. Lauriere C. (2012). Characterization of Arabidopsis calcium-dependent protein kinases: activated or not by calcium? Biochem. J. 447, 291–299. doi: 10.1042/BJ20112072, PMID: 22827269

[ref5] Bredow M. Bender K. W. Dingee A. J. Holmes D. R. Thomson A. Ciren D.. (2021). Phosphorylation-dependent subfunctionalization of the calcium-dependent protein kinase CPK28. Proc. Natl. Acad. Sci. U. S. A. 118:e2024272118. doi: 10.1073/pnas.2024272118, PMID: 33941701 PMC8126791

[ref6] Buscaill P. Rivas S. (2014). Transcriptional control of plant defence responses. Curr. Opin. Plant Biol. 20, 35–46. doi: 10.1016/j.pbi.2014.04.004, PMID: 24840291

[ref7] Chang Y. Li B. Shi Q. Geng R. Geng S. Liu J.. (2020). Comprehensive analysis of respiratory burst oxidase homologs (Rboh) gene family and function of GbRboh5/18 on Verticillium wilt resistance in *Gossypium barbadense*. Front. Genet. 11:788. doi: 10.3389/fgene.2020.00788, PMID: 33061930 PMC7517705

[ref8] Chen H. Zou Y. Shang Y. Lin H. Wang Y. Cai R.. (2008). Firefly luciferase complementation imaging assay for protein-protein interactions in plants. Plant Physiol. 146, 368–376. doi: 10.1104/pp.107.111740, PMID: 18065554 PMC2245818

[ref9] Cheng H. Q. Han L. B. Yang C. L. Wu X. M. Zhong N. Q. Wu J. H.. (2016). The cotton MYB108 forms a positive feedback regulation loop with CML11 and participates in the defense response against *Verticillium dahliae* infection. J. Exp. Bot. 67, 1935–1950. doi: 10.1093/jxb/erw016, PMID: 26873979 PMC4783372

[ref10] Cheng S. H. Willmann M. R. Chen H. C. Sheen J. (2002). Calcium signaling through protein kinases. The Arabidopsis calcium-dependent protein kinase gene family. Plant Physiol. 129, 469–485. doi: 10.1104/pp.005645, PMID: 12068094 PMC1540234

[ref11] Clough S. J. Bent A. F. (1998). Floral dip: a simplified method for Agrobacterium-mediated transformation of *Arabidopsis thaliana*. Plant J. 16, 735–743. doi: 10.1046/j.1365-313x.1998.00343.x, PMID: 10069079

[ref12] Coca M. Segundo B. (2010). AtCPK1 calcium-dependent protein kinase mediates pathogen resistance in Arabidopsis. Plant J. 63, 526–540. doi: 10.1111/j.1365-313X.2010.04255.x, PMID: 20497373

[ref13] Defalco T. A. Zipfel C. (2021). Molecular mechanisms of early plant pattern-triggered immune signaling. Mol. Cell 81, 3449–3467. doi: 10.1016/j.molcel.2021.07.029, PMID: 34403694

[ref14] Ding Y. Sun T. Ao K. Peng Y. Zhang Y. Li X.. (2018). Opposite roles of salicylic acid receptors NPR1 and NPR3/NPR4 in transcriptional regulation of plant immunity. Cell 173, 1454–1467.e15. doi: 10.1016/j.cell.2018.03.044, PMID: 29656896

[ref15] Dubiella U. Seybold H. Durian G. Komander E. Lassig R. Witte C. P.. (2013). Calcium-dependent protein kinase/NADPH oxidase activation circuit is required for rapid defense signal propagation. Proc. Natl. Acad. Sci. U. S. A. 110, 8744–8749. doi: 10.1073/pnas.1221294110, PMID: 23650383 PMC3666735

[ref16] Durner J. Wendehenne D. Klessig D. F. (1998). Defense gene induction in tobacco by nitric oxide, cyclic GMP, and cyclic ADP-ribose. Proc. Natl. Acad. Sci. U. S. A. 95, 10328–10333. doi: 10.1073/pnas.95.17.10328, PMID: 9707647 PMC21508

[ref17] Feng H. J. Li C. Zhou J. L. Yuan Y. Feng Z. L. Shi Y. Q.. (2021). A cotton WAKL protein interacted with a DnaJ protein and was involved in defense against *Verticillium dahliae*. Int. J. Biol. Macromol. 167, 633–643. doi: 10.1016/j.ijbiomac.2020.11.191, PMID: 33275973

[ref18] Fradin E. F. Thomma B. P. H. J. (2006). Physiology and molecular aspects of Verticillium wilt diseases caused by V-dahliae and V-albo-atrum. Mol. Plant Pathol. 7, 71–86. doi: 10.1111/j.1364-3703.2006.00323.x, PMID: 20507429

[ref19] Gao X. Li F. Li M. Kianinejad A. S. Dever J. K. Wheeler T. A.. (2013a). Cotton GhBAK1 mediates Verticillium wilt resistance and cell death. J. Integr. Plant Biol. 55, 586–596. doi: 10.1111/jipb.12064, PMID: 23675706 PMC4395461

[ref20] Gao W. Long L. Zhu L. F. Xu L. Gao W. H. Sun L. Q.. (2013b). Proteomic and virus-induced gene silencing (VIGS) analyses reveal that gossypol, brassinosteroids, and jasmonic acid contribute to the resistance of cotton to *Verticillium dahliae*. Mol. Cell. Proteomics 12, 3690–3703. doi: 10.1074/mcp.M113.031013, PMID: 24019146 PMC3861717

[ref21] Gao X. Q. Wheeler T. Li Z. H. Kenerley C. M. He P. Shan L. B. (2011). Silencing GhNDR1 and GhMKK2 compromises cotton resistance to Verticillium wilt. Plant J. 66, 293–305. doi: 10.1111/j.1365-313X.2011.04491.x, PMID: 21219508 PMC3078967

[ref22] Gong Q. Yang Z. Chen E. Sun G. He S. Butt H. I.. (2018). A phi-class glutathione S-transferase gene for Verticillium wilt resistance in *Gossypium arboreum* identified in a genome-wide association study. Plant Cell Physiol. 59, 275–289. doi: 10.1093/pcp/pcx180, PMID: 29165718

[ref81] Han L. Sun Y. Zhou X. Hao X. Wu M. Zhang X. Feng J. (2021). A novel glycoprotein from Streptomyces sp. triggers early responses of plant defense. Pestic Biochem. Physiol. 171:104719., PMID: 33357541 10.1016/j.pestbp.2020.104719

[ref23] Harper J. F. Sussman M. R. Schaller G. E. Putnam-Evans C. Charbonneau H. Harmon A. C. (1991). A calcium-dependent protein kinase with a regulatory domain similar to calmodulin. Science 252, 951–954. doi: 10.1126/science.1852075, PMID: 1852075

[ref24] He X. Zhu L. Wassan G. M. Wang Y. Miao Y. Shaban M.. (2018). GhJAZ2 attenuates cotton resistance to biotic stresses via the inhibition of the transcriptional activity of GhbHLH171. Mol. Plant Pathol. 19, 896–908. doi: 10.1111/mpp.12575, PMID: 28665036 PMC6638010

[ref25] Hrabak E. M. Chan C. W. Gribskov M. Harper J. F. Choi J. H. Halford N.. (2003). The Arabidopsis CDPK-SnRK superfamily of protein kinases. Plant Physiol. 132, 666–680. doi: 10.1104/pp.102.011999, PMID: 12805596 PMC167006

[ref26] Hu Q. Ao C. Wang X. Wu Y. Du X. (2021). GhWRKY1-like, a WRKY transcription factor, mediates drought tolerance in Arabidopsis via modulating ABA biosynthesis. BMC Plant Biol. 21:458. doi: 10.1186/s12870-021-03238-5, PMID: 34625048 PMC8501554

[ref27] Hu Y. Chen J. Fang L. Zhang Z. Ma W. Niu Y.. (2019). *Gossypium barbadense* and *Gossypium hirsutum* genomes provide insights into the origin and evolution of allotetraploid cotton. Nat. Genet. 51, 739–748. doi: 10.1038/s41588-019-0371-5, PMID: 30886425

[ref28] Hu B. Jin J. P. Guo A. Y. Zhang H. Luo J. C. Gao G. (2015). GSDS 2.0: an upgraded gene feature visualization server. Bioinformatics 31, 1296–1297. doi: 10.1093/bioinformatics/btu817, PMID: 25504850 PMC4393523

[ref29] Hu C. H. Zeng Q. D. Tai L. Li B. B. Zhang P. P. Nie X. M.. (2020). Interaction between TaNOX7 and TaCDPK13 contributes to plant fertility and drought tolerance by regulating ROS production. J. Agric. Food Chem. 68, 7333–7347. doi: 10.1021/acs.jafc.0c02146, PMID: 32551586

[ref30] Hu Q. Zhu L. F. Zhang X. N. Guan Q. Q. Xiao S. H. Min L.. (2018). GhCPK33 negatively regulates defense against *Verticillium dahliae* by phosphorylating GhOPR3. Plant Physiol. 178, 876–889. doi: 10.1104/pp.18.00737, PMID: 30150302 PMC6181045

[ref31] Imami K. Milek M. Bogdanow B. Yasuda T. Kastelic N. Zauber H.. (2018). Phosphorylation of the ribosomal protein RPL12/uL11 affects translation during mitosis. Mol. Cell 72, 84–98.e9. doi: 10.1016/j.molcel.2018.08.019, PMID: 30220558

[ref32] Ivashuta S. Liu J. Y. Liu J. Q. Lohar D. P. Haridas S. Bucciarelli B.. (2005). RNA interference identifies a calcium-dependent protein kinase involved in *Medicago truncatula* root development. Plant Cell 17, 2911–2921. doi: 10.1105/tpc.105.035394, PMID: 16199614 PMC1276019

[ref33] Jin Y. Ye N. H. Zhu F. Y. Li H. X. Wang J. Jiang L. W.. (2017). Calcium-dependent protein kinase CPK28 targets the methionine adenosyltransferases for degradation by the 26S proteasome and affects ethylene biosynthesis and lignin deposition in Arabidopsis. Plant J. 90, 304–318. doi: 10.1111/tpj.13493, PMID: 28112445

[ref34] Kim Y. H. Park S. C. Yun B. W. Kwak S. S. (2017). Overexpressing sweetpotato peroxidase gene swpa4 affects nitric oxide production by activating the expression of reactive oxygen species- and nitric oxide-related genes in tobacco. Plant Physiol. Biochem. 120, 52–60. doi: 10.1016/j.plaphy.2017.09.023, PMID: 28987862

[ref35] Klosterman S. J. Atallah Z. K. Vallad G. E. Subbarao K. V. (2009). Diversity, pathogenicity; and management of Verticillium species. Annu. Rev. Phytopathol. 47, 39–62. doi: 10.1146/annurev-phyto-080508-081748, PMID: 19385730

[ref36] Komatsu S. Yang G. X. Khan M. Onodera H. Toki S. Yamaguchi M. (2007). Over-expression of calcium-dependent protein kinase 13 and calreticulin interacting protein 1 confers cold tolerance on rice plants. Mol. Gen. Genomics. 277, 713–723. doi: 10.1007/s00438-007-0220-6, PMID: 17318583

[ref37] Krzywinski M. Schein J. Birol I. Connors J. Gascoyne R. Horsman D.. (2009). Circos: an information aesthetic for comparative genomics. Genome Res. 19, 1639–1645. doi: 10.1101/gr.092759.109, PMID: 19541911 PMC2752132

[ref38] Lecourieux D. Raneva R. Pugin A. (2006). Calcium in plant defence-signalling pathways. New Phytol. 171, 249–269. doi: 10.1111/j.1469-8137.2006.01777.x, PMID: 16866934

[ref39] Li Y. Fei X. Dai H. Li J. Zhu W. Deng X. (2019a). Genome-wide identification of calcium-dependent protein kinases in *Chlamydomonas reinhardtii* and functional analyses in nitrogen deficiency-induced oil accumulation. Front. Plant Sci. 10:1147. doi: 10.3389/fpls.2019.01147, PMID: 31695707 PMC6818280

[ref40] Li J. Li Y. Deng Y. Chen P. Feng F. Chen W.. (2018). A calcium-dependent protein kinase, ZmCPK32, specifically expressed in maize pollen to regulate pollen tube growth. PLoS One 13:e0195787. doi: 10.1371/journal.pone.0209939, PMID: 29813101 PMC5973587

[ref41] Li B. Meng X. Z. Shan L. B. He P. (2016). Transcriptional regulation of pattern-triggered immunity in plants. Cell Host Microbe 19, 641–650. doi: 10.1016/j.chom.2016.04.011, PMID: 27173932 PMC5049704

[ref42] Li T. G. Wang B. L. Yin C. M. Zhang D. D. Wang D. Song J.. (2019b). The *Gossypium hirsutum* TIR-NBS-LRR gene GhDSC1 mediates resistance against Verticillium wilt. Mol. Plant Pathol. 20, 857–876. doi: 10.1111/mpp.12797, PMID: 30957942 PMC6637886

[ref43] Liese A. Romeis T. (2013). Biochemical regulation of in vivo function of plant calcium-dependent protein kinases (CDPK). BBA-Mol. Cell. Res. 1833, 1582–1589. doi: 10.1016/j.bbamcr.2012.10.024, PMID: 23123193

[ref44] Liu W. Li W. He Q. Daud M. K. Chen J. Zhu S. (2014). Genome-wide survey and expression analysis of calcium-dependent protein kinase in *Gossypium raimondii*. PLoS One 9:e98189. doi: 10.1371/journal.pone.0116352, PMID: 24887436 PMC4041719

[ref45] Liu K. H. Niu Y. Konishi M. Wu Y. Du H. Chung H. S.. (2017). Discovery of nitrate-CPK-NLP signalling in central nutrient-growth networks. Nature 545, 311–316. doi: 10.1038/nature22077, PMID: 28489820 PMC5823009

[ref46] Lu S. N. Wang J. Chitsaz F. Derbyshire M. K. Geer R. C. Gonzales N. R.. (2020). CDD/SPARCLE: the conserved domain database in 2020. Nucleic Acids Res. 48, D265–D268. doi: 10.1093/nar/gkz991, PMID: 31777944 PMC6943070

[ref47] Luna E. Pastor V. Robert J. Flors V. Mauch-Mani B. Ton J. (2011). Callose deposition: a multifaceted plant defense response. Mol. Plant Microbe Interact. 24, 183–193. doi: 10.1094/MPMI-07-10-0149, PMID: 20955078

[ref48] Macho A. P. Zipfel C. (2014). Plant PRRs and the activation of innate immune signaling. Mol. Cell 54, 263–272. doi: 10.1016/j.molcel.2014.03.028, PMID: 24766890

[ref49] Matschi S. Hake K. Herde M. Hause B. Romeis T. (2015). The calcium-dependent protein kinase CPK28 regulates development by inducing growth phase-specific, spatially restricted alterations in jasmonic acid levels independent of defense responses in Arabidopsis. Plant Cell 27, 591–606. doi: 10.1105/tpc.15.00024, PMID: 25736059 PMC4558673

[ref50] Matschi S. Werner S. Schulze W. X. Legen J. Hilger H. H. Romeis T. (2013). Function of calcium-dependent protein kinase CPK28 of *Arabidopsis thaliana* in plant stem elongation and vascular development. Plant J. 73, 883–896. doi: 10.1111/tpj.12090, PMID: 23252373

[ref51] Monaghan J. (2018). Conserved degradation of orthologous RLCKs regulates immune homeostasis. Trends Plant Sci. 23, 554–557. doi: 10.1016/j.tplants.2018.05.001, PMID: 29776748

[ref52] Monaghan J. Matschi S. Shorinola O. Rovenich H. Matei A. Segonzac C.. (2014). The calcium-dependent protein kinase CPK28 buffers plant immunity and regulates BIK1 turnover. Cell Host Microbe 16, 605–615. doi: 10.1016/j.chom.2014.10.007, PMID: 25525792

[ref53] Nagaraj S. Senthil-Kumar M. Ramu V. S. Wang K. R. Mysore K. S. (2016). Plant ribosomal proteins, RPL12 and RPL19, play a role in nonhost disease resistance against bacterial pathogens. Front. Plant Sci. 6:1192. doi: 10.3389/fpls.2015.01192, PMID: 26779226 PMC4702080

[ref54] Nurnberger T. Brunner F. Kemmerling B. Piater L. (2004). Innate immunity in plants and animals: striking similarities and obvious differences. Immunol. Rev. 198, 249–266. doi: 10.1111/j.0105-2896.2004.0119.x, PMID: 15199967

[ref55] Oger E. Marino D. Guigonis J. M. Pauly N. Puppo A. (2012). Sulfenylated proteins in the *Medicago truncatula*-*Sinorhizobium meliloti* symbiosis. J. Proteome 75, 4102–4113. doi: 10.1016/j.jprot.2012.05.024, PMID: 22634402

[ref56] Ranf S. Eschen-Lippold L. Frhlich K. Westphal L. Scheel D. Lee J. (2014). Microbe-associated molecular pattern-induced calcium signaling requires the receptor-like cytoplasmic kinases, PBL1 and BIK1. BMC Plant Biol. 14:374. doi: 10.1186/s12870-014-0374-4, PMID: 25522736 PMC4279983

[ref57] Romeis T. Ludwig A. A. Martin R. Jones J. D. G. (2001). Calcium-dependent protein kinases play an essential role in a plant defence response. EMBO J. 20, 5556–5567. doi: 10.1093/emboj/20.20.5556, PMID: 11597999 PMC125278

[ref58] Shaban M. Miao Y. H. Ullah A. Khan A. Q. Menghwar H. Khan A. H.. (2018). Physiological and molecular mechanism of defense in cotton against *Verticillium dahliae*. Plant Physiol. Biochem. 125, 193–204. doi: 10.1016/j.plaphy.2018.02.011, PMID: 29462745

[ref59] Singh K. Foley R. C. Onate-Sanchez L. (2002). Transcription factors in plant defense and stress responses. Curr. Opin. Plant Biol. 5, 430–436. doi: 10.1016/S1369-5266(02)00289-3, PMID: 12183182

[ref60] Sun Q. Jiang H. Z. Zhu X. Y. Wang W. N. He X. H. Shi Y. Z.. (2013). Analysis of sea-island cotton and upland cotton in response to *Verticillium dahliae* infection by RNA sequencing. BMC Genomics 14:852. doi: 10.1186/1471-2164-14-852, PMID: 24314117 PMC3878982

[ref61] Tan X. P. Liang W. Q. Liu C. J. Luo P. Heinstein P. Chen X. Y. (2000). Expression pattern of (+)-delta-cadinene synthase genes and biosynthesis of sesquiterpene aldehydes in plants of *Gossypium arboreum* L. Planta 210, 644–651. doi: 10.1007/s004250050055, PMID: 10787059

[ref62] Tang Y. Zhang Z. N. Lei Y. Hu G. Liu J. F. Hao M. Y.. (2019). Cotton WATs modulate SA biosynthesis and local lignin deposition participating in plant resistance against *Verticillium dahliae*. Front. Plant Sci. 10:526. doi: 10.3389/fpls.2019.00526, PMID: 31105726 PMC6499033

[ref63] Tewari R. K. Horemans N. Nauts R. Wannijn J. Van Hees M. Vandenhove H. (2019). The nitric oxide suppressed Arabidopsis mutants-Atnoa1 and Atnia1nia2noa1-2 produce nitric oxide in MS growth medium and on uranium exposure. Plant Physiol. Biochem. 140, 9–17. doi: 10.1016/j.plaphy.2019.04.042, PMID: 31078053

[ref64] Vijayakumar P. Datta S. Dolan L. (2016). ROOT HAIR DEFECTIVE SIX-LIKE4 (RSL4) promotes root hair elongation by transcriptionally regulating the expression of genes required for cell growth. New Phytol. 212, 944–953. doi: 10.1111/nph.14095, PMID: 27452638 PMC5111604

[ref65] Wang J. L. Grubb L. E. Wang J. Liang X. Li L. Gao C.. (2018). A regulatory module controlling homeostasis of a plant immune kinase. Mol. Cell 69, 493–504.e6. doi: 10.1016/j.molcel.2017.12.026, PMID: 29358080

[ref66] Wang J. P. Xu Y. P. Munyampundu J. P. Liu T. Y. Cai X. Z. (2016a). Calcium-dependent protein kinase (CDPK) and CDPK-related kinase (CRK) gene families in tomato: genome-wide identification and functional analyses in disease resistance. Mol. Gen. Genomics. 291, 661–676. doi: 10.1007/s00438-015-1137-0, PMID: 26520101

[ref67] Wang W. Yuan Y. Yang C. Geng S. Sun Q. Long L.. (2016b). Characterization, expression, and functional analysis of a novel NAC gene associated with resistance to Verticillium wilt and abiotic stress in cotton. G3 6, 3951–3961. doi: 10.1534/g3.116.034512, PMID: 27784753 PMC5144965

[ref68] Wu S. Y. Zhen C. Y. Wang K. Gao H. Y. (2019). Effects of *Bacillus subtilis* CF-3 VOCs combined with heat treatment on the control of *Monilinia fructicola* in peaches and Colletotrichum gloeosporioides in litchi fruit. J. Food Sci. 84, 3418–3428. doi: 10.1111/1750-3841.14949, PMID: 31762032

[ref69] Xu L. Zhu L. F. Tu L. L. Liu L. L. Yuan D. J. Jin L.. (2011). Lignin metabolism has a central role in the resistance of cotton to the wilt fungus *Verticillium dahliae* as revealed by RNA-Seq-dependent transcriptional analysis and histochemistry. J. Exp. Bot. 62, 5607–5621. doi: 10.1093/jxb/err245, PMID: 21862479 PMC3223054

[ref70] Yang D. H. Hettenhausen C. Baldwin I. T. Wu J. Q. (2012). Silencing *Nicotiana attenuata* calcium-dependent protein kinases, CDPK4 and CDPK5, strongly up-regulates wound- and herbivory-induced jasmonic acid accumulations. Plant Physiol. 159, 1591–1607. doi: 10.1104/pp.112.199018, PMID: 22715110 PMC3425199

[ref71] Yang J. Wang X. F. Xie M. X. Wang G. N. Li Z. K. Zhang Y.. (2020). Proteomic analyses on xylem sap provides insights into the defense response of *Gossypium hirsutum* against *Verticillium dahliae*. J. Proteome 213:103599. doi: 10.1016/j.jprot.2019.103599, PMID: 31809902

[ref72] Yang J. Zhang Y. Wang X. F. Wang W. Q. Li Z. K. Wu J. H.. (2018). HyPRP1 performs a role in negatively regulating cotton resistance to V-dahliae via the thickening of cell walls and ROS accumulation. BMC Plant Biol. 18:339. doi: 10.1186/s12870-018-1565-1, PMID: 30526498 PMC6286592

[ref73] Ye S. Wang L. Xie W. Wan B. Li X. Lin Y. (2009). Expression profile of calcium-dependent protein kinase (CDPKs) genes during the whole lifespan and under phytohormone treatment conditions in rice (*Oryza sativa* L. ssp. *indica*). Plant Mol. Biol. 70, 311–325. doi: 10.1007/s11103-009-9475-0, PMID: 19263224

[ref74] Zhang W. Zhang H. Liu K. Jian G. Qi F. Si N. (2017). Large-scale identification of *Gossypium hirsutum* genes associated with *Verticillium dahliae* by comparative transcriptomic and reverse genetics analysis. PLoS One 12:e0181609. doi: 10.1371/journal.pone.0190180, PMID: 28767675 PMC5540499

[ref75] Zhao P. Liu Y. Kong W. Ji J. Cai T. Guo Z. (2021). Genome-wide identification and characterization of calcium-dependent protein kinase (CDPK) and CDPK-related kinase (CRK) gene families in *Medicago truncatula*. Int. J. Mol. Sci. 22:1044. doi: 10.3390/ijms222111850, PMID: 33494310 PMC7864493

[ref76] Zhao L. N. Shen L. K. Zhang W. Z. Zhang W. Wang Y. Wu W. H. (2013). Ca2+-dependent protein kinase11 and 24 modulate the activity of the inward rectifying K+ channels in Arabidopsis pollen tubes. Plant Cell 25, 649–661. doi: 10.1105/tpc.112.103184, PMID: 23449501 PMC3608784

[ref77] Zhou J. L. Feng Z. L. Liu S. C. Wei F. Shi Y. Q. Zhao L. H.. (2021). CGTase, a novel antimicrobial protein from *Bacillus cereus* YUPP-10, suppresses *Verticillium dahliae* and mediates plant defence responses. Mol. Plant Pathol. 22, 130–144. doi: 10.1111/mpp.13014, PMID: 33230892 PMC7749748

[ref78] Zhu D. D. Zhang X. Zhou J. Wu Y. Zhang X. Feng Z.. (2021). Genome-wide analysis of ribosomal protein GhRPS6 and its role in cotton Verticillium wilt resistance. Int. J. Mol. Sci. 22:1745. doi: 10.3390/ijms22041795, PMID: 33670294 PMC7918698

[ref79] Zuo R. Hu R. Chai G. Xu M. Qi G. Kong Y.. (2013). Genome-wide identification, classification, and expression analysis of CDPK and its closely related gene families in poplar (*Populus trichocarpa*). Mol. Biol. Rep. 40, 2645–2662. doi: 10.1007/s11033-012-2351-z, PMID: 23242656

[ref80] Zuo K. J. Qin J. Zhao J. Y. Ling H. Zhang L. D. Cao Y. F.. (2007). Over-expression GbERF2 transcription factor in tobacco enhances brown spots disease resistance by activating expression of downstream genes. Gene 391, 80–90. doi: 10.1016/j.gene.2006.12.019, PMID: 17321073

