# Supplementary material for: Calcium-Dependent Protein Kinase GhCDPK28 Was Dentified and Involved in Verticillium Wilt Resistance in Cotton
Source: Front Plant Sci. 2021 Dec 15;12:772649. doi: 10.3389/fpls.2021.772649 (PMC8715758; doi:10.3389/fpls.2021.772649)
Supplement: Supplementary file 1 [file Data_Sheet_1.docx]

Supplementary Material


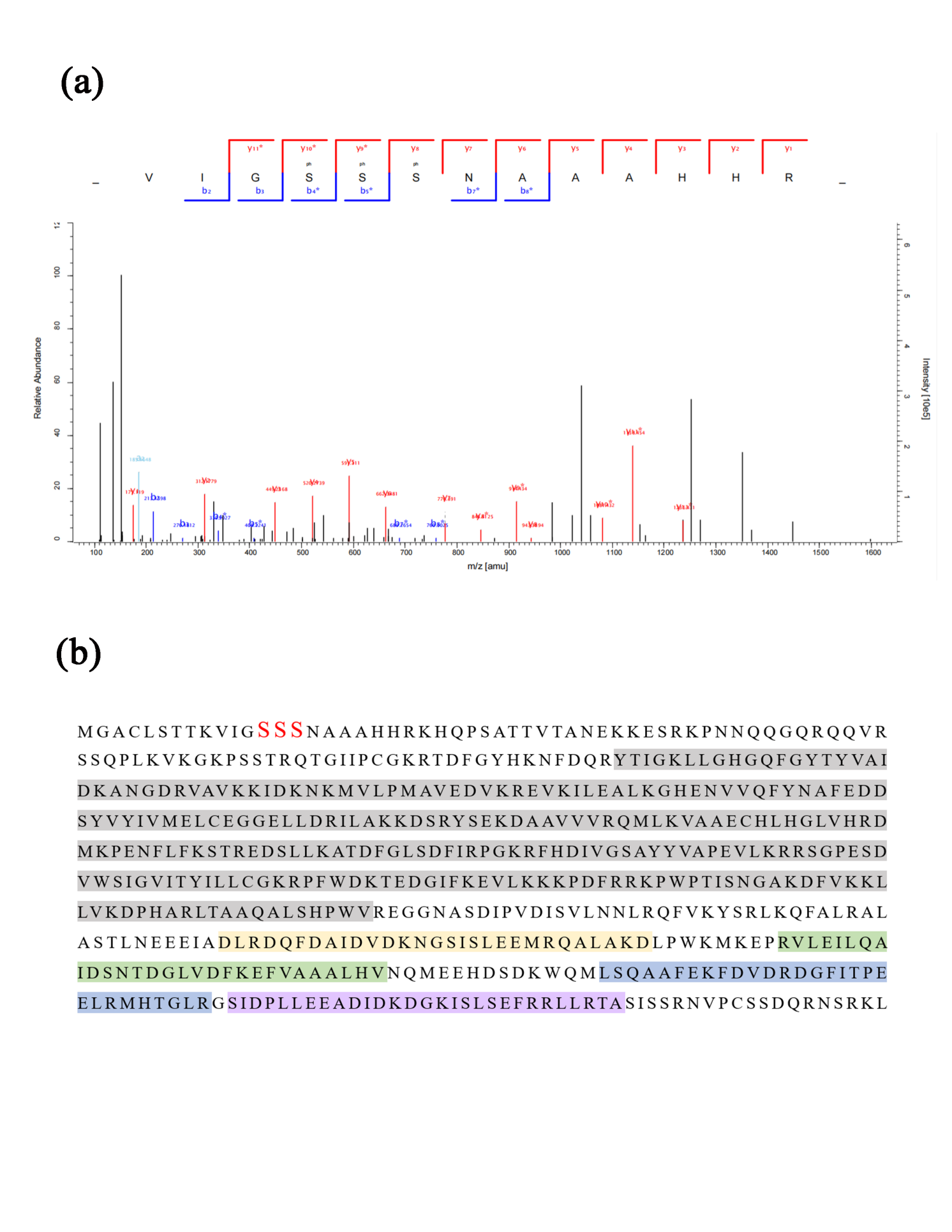


**Supplementary Figure 1.** GhCDPK28-6 was phosphorylated at Ser13, 14 and 15 after inoculation with *V. dahliae.* (a) Mass spectrogram of GhCDPK28-6 phosphorylation after inoculation with *V. dahliae*. (b) Amino acid sequence of GhCDPK28-6. The sites marked in red are serine 13,14,15, and phosphorylation after inoculation；In gray are serine/threonine protein kinases, catalytic domains; Marked yellow, green, blue, and purple are EF hands, calcium binding motifs.


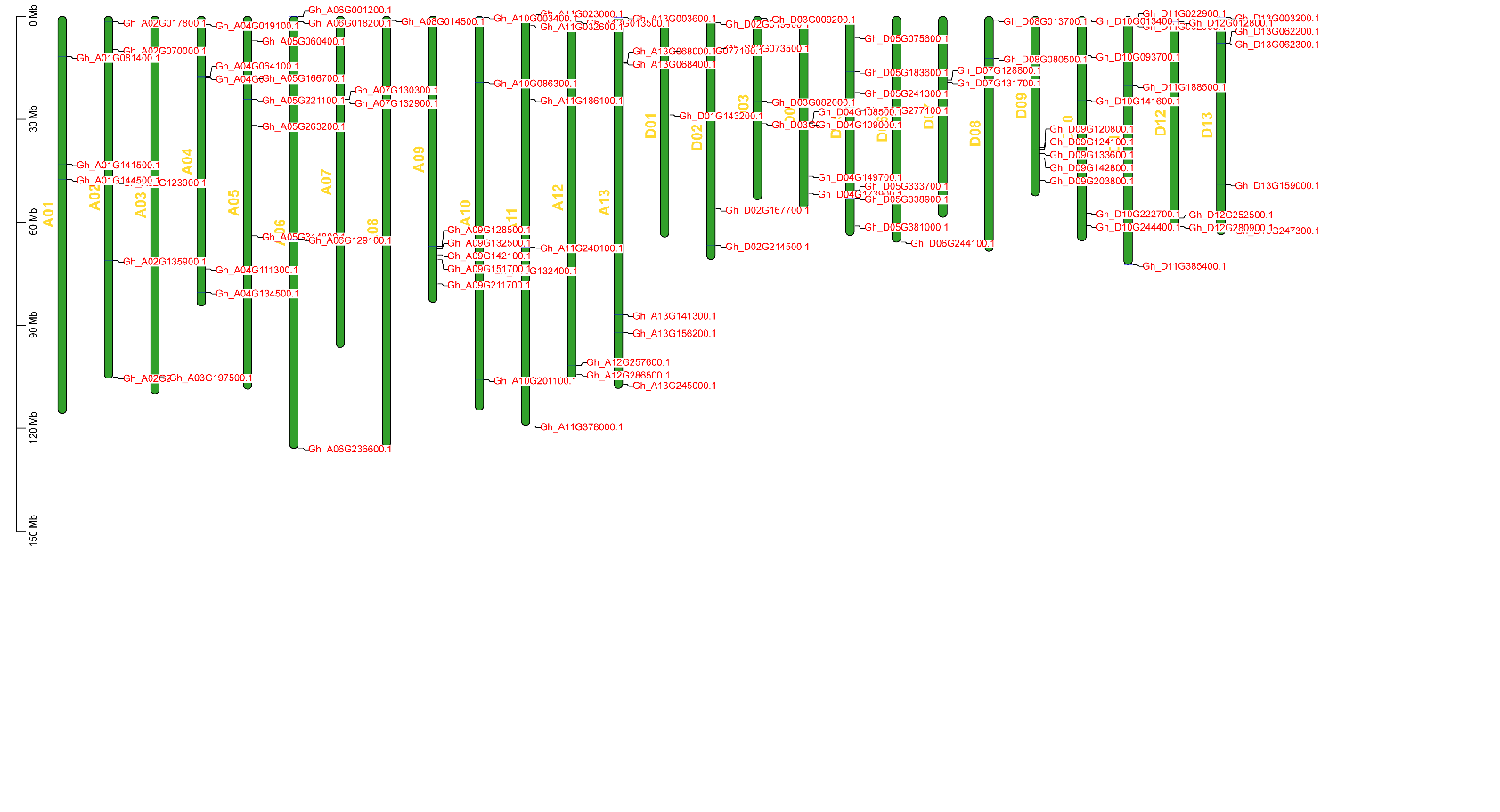


**Supplementary Figure 2.** Chromosomal distribution of *GhCDPK* genes. The genome visualization tool CIRCOS was used to illustrate the chromosomal distribution of *GhCDPK* genes. The chromosome number is shown in yellow letters in the middle of each chromosome. The scale bar beside the chromosome indicates the length in megabases (Mb).


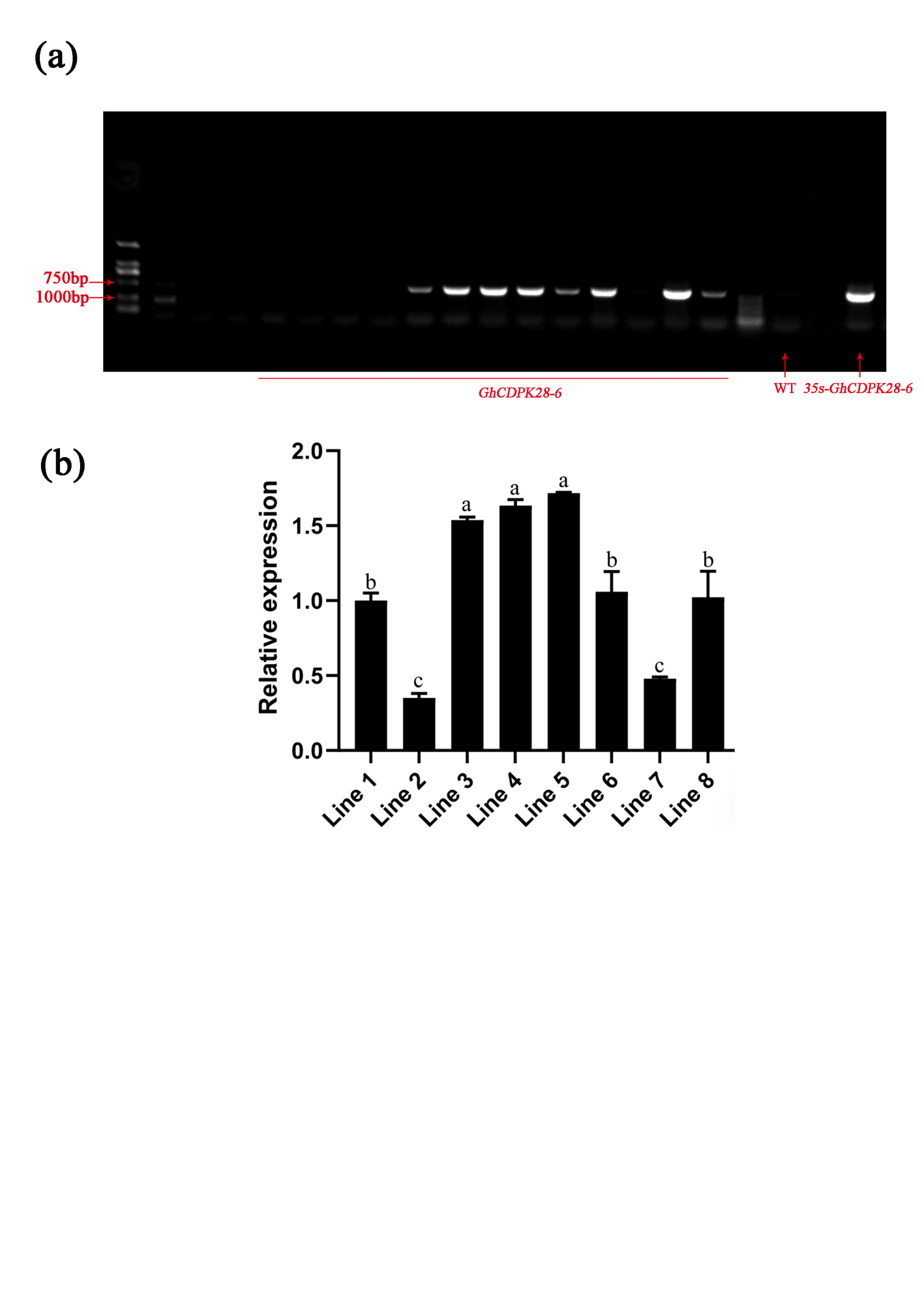


**Supplementary Figure 3.** Identification of transgenic *Arabidopsis thaliana*. (a), PCR detection of transgenic *Arabidopsis thaliana*. (b), Fluorescence quantitative analysis of *GhCDPK28-6* expression in *Arabidopsis thaliana*. Error bars represent the standard deviation of three biological replicates. Different letters indicate significant difference at p < 0.05.

| **Supplementary Tabel 1.** Primers used in this research work. | | |
| --- | --- | --- |
| Primer name | Sequences (5’-3’) | Destination |
| *CDPK28-6-full-F* | ATGGGAGCCTGTCTCTCCACTACCA | Gene cloning |
| *CDPK28-6-full-R* | TAACTTCCGAGAATTCCGCTGATCA |  |
| *CDPK28-6-VIGS-F* | CGGAATTCCGCTAAAAAAGATAGTCGT | VIGS |
| *CDPK28-6-VIGS-R* | GGGGTACCCCAAAGCAAAATGTAGGTAA |  |
| *CDPK28-6-OE-F* | CACGGGGGACTCTAGAATGGGAGCCTGTCTCTCCACT | overexpression |
| *CDPK28-6-OE-R* | TGTTAATTAAGGATCCTAACTTCCGAGAATTCCGCTGA |  |
| *BD-CDPK28-6-F* | CATGGAGGCCGAATTCATGGGAGCCTGTCTCTCCACT | Y2H |
| *BD-CDPK28-6-R* | GCAGGTCGACGGATCCCTATAACTTCCGAGAATTCCGCTGA |  |
| *AD-GhRPL12C-F* | GGAGGCCAGTGAATTCATGCCGCCGAAGTTTGACC |  |
| *AD-GhRPL12C-R* | CGAGCTCGATGGATCCTCCAAGGGAACATCGACTTCC |  |
| *AD-GhPBL9-F* | GGAGGCCAGTGAATTCATGGGGTCTTGTTTTAGTGC |  |
| *AD-GhPBL9-R* | CGAGCTCGATGGATCTGTACTCAATGGAGAAGCAGC |  |
| *GhUbiquitin-F* | GAGTCTTCGGACACCATTG | RT-qPCR for |
| *GhUbiquitin-R* | CTTGACCTTCTTCTTCTTGTGC | endogenous reference |
| *GhNOA-F* | GAGGATGCTGAAAGACCTGCTA | RT-qPCR for *GhNOA* |
| *GhNOA-R* | TCTCAACTGGCTTGGGTACATG |  |
| *GhPR1-F* | ATGATTGAAGGTCGGCCTTTAGGG | RT-qPCR for *GhPR1* |
| *GhPR1-R* | CAGCTGCCACAAACTGGTTCTCAT |  |
| *GhC4H1-F* | CCGAACCCGACACCCATAAGC | RT-qPCR for *GhC4H1* |
| *GhC4H1-R* | GCAGGGATGTCATACCCACCAAG |  |
| *GhPAL-F* | TGGTGGCTGAGTTTAGGAAA | RT-qPCR for *GhPAL* |
| *GhPAL-R* | TGAGTGAGGCAATGTGTGA |  |
| *GhPPO-F* | CCGCATAACCATCACAAG | RT-qPCR for *GhPPO* |
| *GhPPO-R* | ACTCTCATCACCTTCAACA |  |
| *GhNPR1-F* | GGCTTGCGGAGAAGACGAC | RT-qPCR for *GhNPR1* |
| *GhNPR1-R* | ACGACGATGAGAGAGTTTACGG |  |
| *qPCR-CDPK28-6-F* | ACTTGCCCACCTTGCCTTTG | RT-qPCR for *GhCDPK28* |
| *qPCR-CDPK28-6-R* | TGCCATGTCTGCACGTTGCT |  |
| *GhCDPK28-6-F* | TGGTGGAGCACGACA | PCR detection of |
| *GhCDPK28-6-R* | AACAGCCATTGGAAGAA | transgenic Arabidopsis |
| *AtPR1-F* | GAGAAGGCTAACTACAACTACGCTG | RT-qPCR for *AtPR1* |
| *AtPR1-R* | ACACCTCACTTTGGCACATCC |  |
| *AtNPR1-F* | CTTTCTAAAACCGTGGAACTCG | RT-qPCR for *AtNPR1* |
| *AtNPR1-R* | GTCGTTTCTCAGCAGTGTCGT |  |
| *AtC4H1-F* | AAACAACCCCAACAGCTGGAA | RT-qPCR for *AtC4H1* |
| *AtC4H1-R* | CACTTTAGACTGTCCTGGAGGAGG |  |
| *AtNOA-F* | TTCTCTTCCTCGTCGCCAC | RT-qPCR for *AtNOA* |
| *AtNOA-R* | CGGATTAGCTCCAACTAAATCAC |  |
| *AtPAL-F* | CTTATTAGATTCCTTAACGCCG | RT-qPCR for *AtPAL* |
| *AtPAL-R* | TCTCCGGAGGCGGTGATTGTA |  |
| *AT UBQ10-F* | AACTTTGGTGGTTTGTGTTTTGG | RT-qPCR for |
| *AT UBQ10-R* | TCGACTTGTCATTAGAAAGAAAGAGATAA | endogenous reference |
| *cLUC-CDPK28-6-F* | \| GTACGCGTCCCGGGGCGGTACCATGGGAGCCTGTCTCTC \| \| --- \| | LCI |
| *cLUC-CDPK28-6-R* | \| GAACGAAAGCTCTGCAGGTCGACCTATAACTTCCGAGAA \| \| --- \| | LCI |
| *nLUC-RPL12C-F* | \| CGGGGGACGAGCTCGGTACCATGCCGCCGAAGTTTGACC \| \| --- \| | LCI |
| *nLUC-RPL12C-R* | \| GTCCCGGGGCGTCGACCTCCAAGGGAACATCGACT \| \| --- \| | LCI |
| *nLUC-PBL9-F* | \| CGGGGGACGAGCTCGGTACCATGGGGTCTTGTTTTAGTG \| \| --- \| | LCI |
| *nLUC-PBL9-R* | \| GTCCCGGGGCGTCGACTGTACTCAATGGAGAAGCA \| \| --- \| | LCI |

| **Supplementary Tabel 2.** Details of the cotton CDPK proteins physiochemical properties | | | | |
| --- | --- | --- | --- | --- |
| **Gene ID** | **Protein Length** | **Molecular WeiGht (kDa)** | **Charge** | **Isoelectric Point** |
| Gorai.007G194500 | 554 | 62.789 | 18.5 | 9.481 |
| Gh_D11G188500 | 554 | 62.789 | 18.5 | 9.481 |
| Gh_A11G186100 | 554 | 62.701 | 18.5 | 9.481 |
| Ga11G2146 | 554 | 62.701 | 18.5 | 9.481 |
| Gorai.011G098300 | 544 | 61.969 | 13 | 8.975 |
| Gh_D10G093700 | 544 | 61.933 | 12 | 8.807 |
| Gh_A10G086300 | 544 | 61.922 | 12 | 8.628 |
| Ga10G1991 | 544 | 62 | 11.5 | 8.627 |
| Gorai.003G009500 | 541 | 61.274 | 13.5 | 9.137 |
| Gh_D03G009200 | 541 | 61.286 | 15.5 | 9.364 |
| Gh_A02G202200 | 541 | 61.334 | 14.5 | 9.256 |
| Ga02G0093 | 541 | 61.268 | 15.5 | 9.364 |
| Gh_A12G286500 | 510 | 56.305 | -7 | 5.608 |
| Ga12G0176 | 510 | 56.265 | -7 | 5.608 |
| Gh_D12G280900 | 510 | 56.196 | -7 | 5.608 |
| Gorai.008G280900 | 510 | 56.196 | -7 | 5.608 |
| Gorai.006G210300 | 509 | 55.642 | -5 | 5.888 |
| Gh_D09G203800 | 509 | 55.684 | -6 | 5.739 |
| Gh_A09G211700 | 477 | 52.192 | -2.5 | 6.192 |
| Ga09G2162 | 510 | 55.888 | -4 | 6.027 |
| Gh_A06G018200 | 96 | 11.166 | 2 | 9.433 |
| Ga06G0198 | 96 | 11.189 | 3 | 9.749 |
| Gh_D08G080500 | 145 | 16.231 | -11 | 4.173 |
| Gh_D02G167700 | 178 | 20.272 | -17.5 | 4.13 |
| Gh_D05G338900 | 183 | 20.813 | -18.5 | 4.128 |
| Gh_D10G244400 | 183 | 20.813 | -18.5 | 4.128 |
| Gorai.013G064400 | 487 | 54.636 | -16 | 4.856 |
| Gh_D13G062300 | 528 | 58.965 | -14 | 4.98 |
| Gh_A13G068400 | 487 | 54.625 | 16 | 4.856 |
| Ga14G1948 | 528 | 58.954 | -14 | 4.98 |
| Gorai.013G064500 | 523 | 58.306 | -1.5 | 6.368 |
| Gh_D13G062200 | 523 | 58.401 | -1 | 6.411 |
| Gh_A13G068000 | 523 | 58.556 | 4.5 | 6.976 |
| Ga13G0324 | 523 | 58.497 | 3.5 | 6.86 |
| Gorai.008G013700 | 575 | 64.604 | -5.5 | 6.066 |
| Gh_D12G012800 | 575 | 64.574 | -5.5 | 6.066 |
| Gh_A12G013500 | 560 | 62.564 | -7.5 | 5.809 |
| Ga12G2790 | 550 | 61.517 | -10.5 | 5.471 |
| Gorai.005G216500 | 568 | 63.6 | -6.5 | 5.919 |
| Gh_D02G214500 | 568 | 63.542 | -5.5 | 6.021 |
| Gh_A03G197500 | 568 | 63.616 | -4.5 | 6.118 |
| Ga03G2256 | 529 | 59.123 | -6.5 | 5.911 |
| Gorai.012G138900 | 552 | 61.958 | -13 | 5.128 |
| Ga04G0151 | 552 | 61.903 | -8 | 5.621 |
| Gh_A04G134500 | 547 | 61.354 | -2 | 6.312 |
| Gh_D04G173900 | 547 | 61.457 | -8 | 5.619 |
| Gorai.009G290200 | 538 | 60.418 | 1.5 | 6.682 |
| Gh_D05G277100 | 502 | 56.401 | -8 | 5.417 |
| Gh_A05G263200 | 502 | 56.467 | -7.5 | 5.531 |
| Ga05G2939 | 502 | 56.467 | -7.5 | 5.531 |
| Gorai.005G074300 | 508 | 56.858 | -8.5 | 5.505 |
| Gh_D02G073500 | 508 | 56.842 | -8.5 | 5.505 |
| Gh_A02G070000 | 511 | 57.143 | -8.5 | 5.508 |
| Ga03G0692 | 511 | 57.143 | -8.5 | 5.508 |
| Gorai.006G147600 | 611 | 68.356 | -16.5 | 4.962 |
| Gh_D09G142800 | 610 | 68.214 | -16.5 | 4.963 |
| Gh_A09G151700 | 610 | 68.242 | -20 | 4.846 |
| Ga09G1513 | 610 | 68.224 | -20 | 4.846 |
| Gorai.002G153600 | 591 | 66.337 | -13.5 | 5.181 |
| Gh_D01G143200 | 591 | 66.268 | -14.5 | 5.121 |
| Gh_A01G144500 | 590 | 66.238 | -12.5 | 5.243 |
| Ga01G1503 | 590 | 66.238 | -12.5 | 5.243 |
| Gorai.009G394700 | 648 | 71.846 | -14 | 5.131 |
| Gh_D04G108500 | 648 | 71.828 | -14 | 5.131 |
| Gh_A04G064100 | 631 | 69.76 | -9 | 5.487 |
| Ga04G1136 | 648 | 71.727 | -13 | 5.189 |
| Gorai.009G395400 | 572 | 63.558 | -13.5 | 5.096 |
| Gh_D04G109000 | 572 | 63.6 | -12.5 | 5.166 |
| Gh_A04G064600 | 399 | 44.883 | -17 | 4.729 |
| Ga04G1139 | 572 | 63.54 | -12.5 | 5.160 |
| Gorai.013G003100 | 579 | 64.625 | -7 | 5.846 |
| Gh_D13G003200 | 573 | 64.085 | -3.5 | 6.216 |
| Gh_A13G003600 | 579 | 64.645 | -5 | 6.055 |
| Ga13G0033 | 579 | 64.601 | -8 | 5.733 |
| Gorai.011G228500 | 587 | 65.36 | -13 | 5.143 |
| Gh_D10G222700 | 583 | 64.898 | -13 | 5.140 |
| Gh_A10G201100 | 587 | 65.542 | -11 | 5.290 |
| Ga10G0671 | 578 | 64.42 | -12.5 | 5.205 |
| Gh_A06G129100 | 202 | 23.896 | 11 | 9.537 |
| Ga06G1308 | 118 | 13.818 | 10.5 | 10.656 |
| Ga02G0781 | 107 | 12.122 | 6 | 10.278 |
| Ga07G1815 | 163 | 18.374 | 5.5 | 9.990 |
| Gh_A09G110200 | 64 | 6.726 | 3 | 10.210 |
| Ga10G1895 | 203 | 22.265 | 11.5 | 10.578 |
| Gh_A13G141300 | 185 | 21.215 | 9 | 10.126 |
| Ga13G1558 | 185 | 21.231 | 9 | 10.126 |
| Gh_A01G141500 | 143 | 16.056 | 11.5 | 10.684 |
| Ga01G1270 | 124 | 14.194 | 11 | 10.721 |
| Gh_A09G132500 | 531 | 59.857 | 0.5 | 6.583 |
| Ga09G1306 | 531 | 59.857 | 0.5 | 6.583 |
| Gorai.006G128200 | 531 | 59.847 | 1 | 6.656 |
| Gh_D09G124100 | 531 | 59.848 | 0 | 6.524 |
| Gh_A11G240100 | 145 | 16.658 | 5 | 8.791 |
| Ga11G1551 | 184 | 21.285 | 18.5 | 10.472 |
| Gh_A07G132900 | 527 | 59.502 | 4.5 | 7.068 |
| Gh_D07G131700 | 526 | 59.535 | 8 | 7.685 |
| Gorai.001G138000 | 529 | 59.459 | 6.5 | 7.403 |
| Ga07G1372 | 529 | 59.372 | 4.5 | 7.057 |
| Gorai.006G137800 | 531 | 60.216 | 2 | 6.756 |
| Gh_ D09G133600 | 531 | 60.228 | 2 | 6.756 |
| Gh_ A09G142100 | 531 | 60.203 | 4 | 7.030 |
| Ga09G1410 | 531 | 60.202 | 4 | 7.030 |
| Gorai.003G092900 | 535 | 60.552 | 4 | 6.861 |
| Gh_ D03G082000 | 550 | 62.29 | 6 | 7.068 |
| Gh_ A02G135900 | 535 | 60.701 | -0.5 | 6.474 |
| Ga02G0762 | 544 | 61.741 | 0 | 6.517 |
| Gorai.005G019800 | 532 | 60.714 | -8 | 5.457 |
| Gh_ D02G019900 | 532 | 60.747 | -9 | 5.356 |
| Gh_ A02G017800 | 532 | 60.713 | -6 | 5.709 |
| Ga03G0178 | 455 | 51.97 | -11 | 5.018 |
| Ga03G0179 | 108 | 12.702 | -4.5 | 4.984 |
| Gorai.011G146000 | 179 | 20.584 | 17.5 | 10.257 |
| Gh_ D10G141600 | 501 | 57.28 | -9 | 5.424 |
| Gh_ A10G132400 | 537 | 61.31 | -4.5 | 6.031 |
| Ga10G1609 | 537 | 61.323 | -4.5 | 6.031 |
| Gorai.009G351200 | 534 | 60.717 | 6.5 | 7.242 |
| Gh_ D05G333700 | 534 | 60.698 | 5 | 7.003 |
| Gh_ A05G314800 | 534 | 60.699 | 5.5 | 7.095 |
| Ga05G3660 | 534 | 60.699 | 5.5 | 7.095 |
| Gorai.007G035100 | 550 | 62.284 | 0 | 6.526 |
| Gh_ D11G032900 | 550 | 62.312 | 0 | 6.526 |
| Gh_ A11G032600 | 550 | 62.326 | 0 | 6.526 |
| Ga11G3781 | 550 | 62.321 | 0.5 | 6.584 |
| Gorai.007G378700 | 527 | 59.245 | -1 | 6.393 |
| Gh_ D11G385400 | 527 | 59.299 | 0 | 6.522 |
| Gh_ A11G378000 | 527 | 59.385 | 0 | 6.521 |
| Ga11G0017 | 527 | 59.353 | -3 | 6.131 |
| Gorai.012G114600 | 527 | 59.435 | -1 | 6.393 |
| Gh_ D04G149700 | 527 | 59.443 | -1 | 6.393 |
| Gh_ A04G111300 | 527 | 59 | -1 | 6.393 |
| Ga04G0619 | 527 | 59.394 | -1 | 6.393 |
| Gorai.011G014200 | 551 | 61.955 | -2 | 6.166 |
| Gh_ D10G013400 | 551 | 62.015 | -2 | 6.166 |
| Gh_ A10G003400 | 552 | 62.205 | 0 | 6.533 |
| Ga10G2961 | 619 | 69.998 | 14.5 | 8.754 |
| Gorai.010G252400 | 527 | 59.318 | -10 | 5.232 |
| Gh_ D06G244100 | 527 | 59.318 | -10 | 5.232 |
| Gh_ A06G236600 | 569 | 64.637 | -11 | 5.248 |
| Ga06G2368 | 543 | 61.274 | -9.5 | 5.337 |
| Gorai.009G438300 | 524 | 58.735 | -0.5 | 6.467 |
| Gh_ D05G381000 | 524 | 58.701 | -0.5 | 6.467 |
| Gh_ A04G019100 | 524 | 58.824 | -1.5 | 6.360 |
| Ga05G4117 | 524 | 58.829 | -1 | 6.418 |
| Gh_ A13G156200 | 536 | 60.367 | -2 | 6.316 |
| Ga13G1730 | 602 | 68.492 | 7.5 | 7.211 |
| Gh_ D13G159000 | 536 | 60.446 | -1 | 6.417 |
| Gorai.013G159300 | 536 | 60.428 | -1.5 | 6.374 |
| Gorai.002G088800 | 537 | 60.458 | 0.5 | 6.565 |
| Gh_ D01G077100 | 537 | 60.46 | 0.5 | 6.565 |
| Gh_ A01G081400 | 537 | 60.536 | -0.5 | 6.470 |
| Ga01G0861 | 598 | 67.208 | 0.5 | 6.558 |
| Gh_ A06G001200 | 526 | 59.241 | -1.5 | 6.340 |
| Ga06G0014 | 526 | 59.246 | -2.5 | 6.217 |
| Gorai.010G001300 | 526 | 59.088 | -2 | 6.262 |
| Gorai.009G191500 | 873 | 97.1 | 3 | 6.707 |
| Gh_ D05G183600 | 907 | 101.033 | 7 | 6.924 |
| Gh_ A05G166700 | 906 | 100.833 | 8 | 6.994 |
| Ga05G1945 | 907 | 100.88 | 8.5 | 7.009 |
| Ga12G1791 | 150 | 16.882 | 8 | 9.970 |
| Ga11G1706 | 163 | 18.467 | 10 | 9.571 |
| Gh_ A05G221100 | 165 | 18.318 | 11 | 10.323 |
| Ga05G2516 | 166 | 18.635 | 10 | 10.199 |
| Ga01G0531 | 158 | 17.595 | 8 | 9.645 |
| Gh_ D05G241300 | 148 | 17.098 | 1.5 | 7.015 |
| Gorai.009G251300 | 148 | 17.126 | 2.5 | 7.459 |
| Ga05G2596 | 117 | 13.561 | -5 | 4.718 |
| Gorai.007G025000 | 530 | 60.385 | 1.5 | 6.661 |
| Gh_ D11G022900 | 431 | 48.705 | 5 | 7.187 |
| Gh_ A11G023000 | 530 | 60.395 | 3.5 | 6.870 |
| Ga11G3891 | 459 | 52.643 | -5.5 | 5.807 |
| Gh_ A12G257600 | 534 | 60.24 | -2.5 | 6.280 |
| Ga12G0469 | 534 | 60.249 | -2 | 6.337 |
| Gh_ D12G252500 | 534 | 60.243 | -1.5 | 6.388 |
| Gorai.008G251000 | 534 | 60.343 | -1.5 | 6.388 |
| Gorai.003G084000 | 508 | 57.547 | 1.5 | 6.681 |
| Gh_ D03G089500 | 509 | 57.362 | -1.5 | 6.338 |
| Gh_ A02G123900 | 519 | 58.6 | -6.5 | 5.768 |
| Ga01G1711 | 519 | 58.711 | -5 | 5.924 |
| Gh_ A13G245000 | 527 | 58.761 | -3.5 | 6.719 |
| Ga13G2664 | 527 | 58.83 | -1.5 | 6.375 |
| Gorai.013G253100 | 527 | 58.792 | -1 | 6.426 |
| Gh_ D13G247300 | 527 | 58.792 | -1 | 6.426 |
| Gorai.004G015100 | 538 | 60.512 | -13 | 5.101 |
| Gh_ D08G013700 | 538 | 60.512 | -13 | 5.101 |
| Gh_ A08G014500 | 538 | 60.494 | -11 | 5.241 |
| Ga08G0159 | 539 | 60.479 | -12 | 5.162 |
| Gorai.001G135000 | 513 | 57.338 | -13.5 | 5.142 |
| Gh_ D07G128800 | 503 | 56.249 | -12.5 | 5.211 |
| Gh_ A07G130300 | 513 | 57.261 | -13.5 | 5.144 |
| Ga07G1339 | 505 | 56.376 | -14.5 | 5.075 |
| Gorai.006G124800 | 524 | 58.594 | -8.5 | 5.521 |
| Gh_ D09G120800 | 524 | 58.566 | -9.5 | 5.408 |
| Gh_ A09G128500 | 523 | 58.551 | -5.5 | 5.899 |
| Ga09G1269 | 523 | 58.552 | -6.5 | 5.773 |
| Gorai.009G078000 | 532 | 59.506 | -12 | 5.314 |
| Gh_ D05G075600 | 533 | 59.7 | -10 | 5.504 |
| Gh_ A05G060400 | 532 | 59.519 | -12 | 5.314 |
| Ga05G0791 | 532 | 59.519 | -12 | 5.314 |
|  |  |  |  |  |
